# Analysis of ICU resistome dynamics in patients, staff and environment for the identification of predictive biomarkers of sepsis and early mortality

**DOI:** 10.1038/s41598-025-10848-8

**Published:** 2025-07-11

**Authors:** Maja Mikolas, Peter Fauszt, Annamaria Petrilla, Peter Nemeth, Peter David, Emese Szilagyi-Tolnai, Anna Szilagyi-Racz, Aniko Stagel, Ferenc Gal, Kristof Gal, Reka Sohajda, Zsombor Szoke, Syed Akib Hossain, Laszlo Stundl, Sandor Biro, Judit Remenyik, Melinda Paholcsek

**Affiliations:** 1 Faculty of Agricultural and Food Sciences and Environmental Management, University of Debrecen, Complex Systems and Microbiome-innovations Centre, Debrecen, Hungary; 2 Central Anesthesiology and Intensive Patient Care Department, Vas County Markusovszky University Teaching Hospital, Szombathely, Hungary; 3 Hungarian National Blood Transfusion Service Nucleic Acid Testing Laboratory, Budapest, Hungary; 4 Department of Oncoradiology, University of Debrecen Clinical Centre, Debrecen, Hungary; 5 Department of Mathematics and Natural Sciences, BRAC University, Dhaka, Bangladesh; 6 Faculty of Agricultural and Food Sciences and Environmental Management, University of Debrecen, Institute of Food Technology, Debrecen, Hungary; 7 Faculty of Medicine, Department of Human Genetics, University of Debrecen, Debrecen, Hungary

**Keywords:** Infectious diseases, Biomarkers, Microbial communities, Pathogens

## Abstract

Antimicrobial resistance (AMR) is a global crisis, posing a critical challenge to healthcare systems, particularly in intensive care units (ICUs), where multidrug-resistant organisms (MDROs) threaten patient survival. This study offers a unique, real-world perspective on AMR dynamics by analyzing 96 metagenomic samples from three key sources: oropharyngeal and rectal swabs of deceased ICU patients (both postadmission and antemortem), healthcare workers, and high-touch ICU surfaces. Findings revealed the ICU environment as a major AMR reservoir, with oropharyngeal swabs carrying the highest AMR burden. While healthcare staff facilitated MDRO spread, they were not primary sources. Staff microbiomes’ MDRO pattern closely resembled environmental samples. Key AMR species included *B. fragilis*, *E. coli*, *S. pneumoniae*, *S. aureus*, with *P. aeruginosa* persisting on high-touch surfaces. Tetracycline resistance was the most prevalent, with common resistances comprising 36.1% of all detected AMR markers. Staff microbial community exhibited higher resistance to macrolides, fluoroquinolones, lincosamides, and cephamycins. A 10-day survival threshold distinguished early (EM) and late mortality (LM) groups. EM patients exhibited unique AMR species in the oropharynx, suggesting respiratory-driven infections, while LM patients showed greater gut-associated resistance. Higher rectal AMR counts correlated with prolonged survival. Notably, four key MDROs (*L. monocytogenes*,* M. tuberculosis*,* S. haemolyticus*, and *S. agalactiae*) were enriched in sepsis patients, suggesting early risk markers. Fewer new resistances emerged in rectal than oropharyngeal swabs, likely due to antibiotic selection pressure. Vancomycin and levofloxacin, frequently co-administered, exerted stronger selective pressure in the oropharynx, possibly explaining the high MRSA prevalence in patient and environmental samples.

**Supplementary Information:**

The online version contains supplementary material available at 10.1038/s41598-025-10848-8.

## Introduction

Healthcare-associated infections (HAIs) pose a major public health challenge, particularly in intensive care units (ICUs), where patients are often critically ill, immunocompromised, and exposed to invasive procedures^1,2^. As a result, morbidity rates in ICUs are 5–10 times higher than in other hospital departments^3^, with studies showing that HAIs affect 9–37% of ICU patients^4^. According to the European Centre for Disease Prevention and Control (ECDC), approximately 3.5 million HAIs occur annually across the EU/EEA, leading to over 90,000 deaths, a burden that exceeds that of influenza and tuberculosis combined^2^. HAIs also account for 71% of infections caused by antibiotic-resistant bacteria, including those resistant to last-line treatments such as carbapenem-resistant *Enterobacterales*^2^. Infections caused by multidrug-resistant organisms (MDROs) result in prolonged hospital stays, increased treatment costs, and severe disease progression^5,6^. Furthermore, up to 50% of HAIs are considered preventable through the implementation of effective infection prevention and control measures^2^. Despite this, an estimated 5% of hospital patients suffer from HAIs, while receiving treatment^7^.

Eubiotic microbiota can protect against external pathogens colonization through a mechanism known as colonization resistance^8^. However, in critical illness, antibiotics can disturb the native intestinal microbiota, leading to the overgrowth of potentially harmful pathogens^9,10^. The progression of infections accelerates due to dysbiotic microbiomes leading to different levels of severity in developing diseases characterized by distinct microbial changes^11,12^.

Within just a few hours of admission, transient microbial species can be detected alongside the typical members of a patient’s microbiota^12,13^. Studying transient microbiota is essential because horizontal gene transfer among these species can lead to commensal bacteria acquiring antibiotic-resistant genes, causing life-threatening nosocomial infections^14,15^.

Globally, approximately 4.95 million people die each year due to infections that cannot be treated with antibiotics^16^. In Europe, over 30,000 people suffer from such infections annually, which accounts for only 0.6% of all cases worldwide, but it could be reduced with a better understanding of the problem^17,18^. The rapid emergence of antibiotic-resistant pathogens in contemporary ICUs is a serious problem nowadays^6^.

Critically ill patients frequently receive systemic antibiotics to suppress or eliminate potential pathogens^19^. However, these interventions, along with altered nutritional intake and the physiological stress of critical illness, can significantly disrupt the oropharyngeal and intestinal microbiota^13,20^. Prolonged antibiotic use reduces microbial diversity, alters the gut metabolome, impairs intestinal defenses, and promotes antibiotic resistance^21^. These changes can lead to increased intestinal permeability, allowing pathogens and microbial products to translocate into the bloodstream, contributing to endotoxemia and potentially affecting distant organs^21^. The gut and oral microbiota are closely connected through the gastrointestinal tract, and dysbiosis in one site may influence the other^21^. Studies suggest that oral pathogens can migrate to the gut and trigger inflammation, while gut dysbiosis may also disturb the oral microbial environment^21^. These interconnected effects are particularly relevant in critically ill patients, where systemic imbalances can exacerbate both local and systemic disease processes.

Strikingly, up to half of ICU patients receive empirical antibiotic therapy without a definitively confirmed infection^5^. Selection pressure and insufficient control of cross-colonization with MDROs can lead to adverse clinical outcomes, and elevate care costs, making the ICU a critical contributor to the spread of these pathogens^5^.

Although data on ICU mortality remains limited, studies suggest that up to 20% of all ICU deaths may occur within the first 24 h, highlighting the need for improved triage and admission strategies to optimize resource use and patient outcomes^22^. While some studies have explored microbiome-based biomarkers for early mortality in ICU patients, most have focused exclusively on the gut microbiome. Research has shown that reduced gut microbial diversity and an increased presence of pathogenic bacteria, such as *Enterobacteriaceae* and Gram-positive anaerobic cocci (GPACs), correlate with higher mortality risk^23^. Furthermore, studies have also shown association between oral microbial diversity and risk of mortality^24–26^. Additionally, antimicrobial resistance (AMR), particularly to β-lactams, glycopeptides, and carbapenems, further complicates patient prognosis^27^. Research has also reported an over 40% mortality rate linked to specific MDROs in ICU patients, like *Acinetobacter baumannii*, *Methicillin-resistant Staphylococcus aureus* (MRSA) and *Vancomycin-resistant Enterococcus* (VRE)^28^. Despite these findings, more research is needed to explore microbiome-based biomarkers beyond the gut, which could enhance early mortality prediction and improve patient management in the ICU.

Sepsis is a leading global cause of death, responsible for 48.9 million cases and 11 million deaths annually^21,22^. It poses a significant burden on healthcare systems, with 15 out of every 1000 hospitalized patients developing sepsis and treatment costs exceeding $32,000 per patient in high-income countries^29,30^. Most microbiome research in sepsis has focused on the gut, revealing significant dysbiosis, loss of diversity, and increased inflammatory and pathogenic bacteria^31,32^. While these findings have provided valuable insights, the role of the oral microbiome remains largely understudied. Given that the oral cavity serves as both a reservoir and a potential entry point for pathogens, its microbiota may play a crucial role in infection dynamics^33^. Oral microbiota or specific taxa have also been linked to systemic inflammation and extra-oral infections, further emphasizing its potential clinical relevance^34^. These insights have led to growing interest in using oral microbiome profiles, frequently collected via non-invasive methods like oral swabs, saliva, or mucosal rinses, as diagnostic or prognostic tools for systemic diseases^34^. Notably, oral swabs have demonstrated greater sensitivity than fecal samples in detecting microbial biomarkers in some conditions^34^. Based on these findings, profiling the oral microbiome in critically ill patients through minimally invasive sampling could be a promising approach for discovering novel biomarkers of sepsis susceptibility, progression, or outcome.

Our study builds upon our previously published article^35^, while there is partial overlap in the patient cohort, the present work significantly extends the analysis by including ICU staff members and environmental samples. Moreover, the current study is focusing specifically on antimicrobial resistance and associated biomarkers, in contrast to the broader microbiome characterization conducted earlier.

While it is well established that ICUs are hotspots for MDRO colonization, and that critically ill patients often receive empirical broad-spectrum antibiotic therapy, placing their microbiomes under extreme stress and increasing the risk of dysbiosis, sepsis, or poor outcomes, most studies to date have only examined these phenomena in isolation^36–39^.

Our study’s uniqueness lies in that it addresses the real-time dynamics of AMR transmission under actual ICU conditions. Specifically, we aimed to quantify the extent of resistome shifts during ICU stay by comparing microbiomes sampled upon admission and throughout hospitalization, using a high-resolution sampling strategy across two anatomically and functionally interconnected microbiomes: the gut and the oropharynx. Despite their physiological links, the interplay between these two microbiomes under critical illness remains underexplored, largely due to the logistical challenges of repeated, multi-site sampling in overburdened ICU environments^13,21^. Yet, in our study, we achieved systematic sampling every three days from both body sites, even in a high-pressure clinical setting.

Moreover, our study is among the very few that integrate patient, staff, and environmental microbiome and resistome data in a comprehensive transmission model. We uniquely considered not only patients and their microbiomes but also those of ICU personnel, specifically their oropharyngeal and rectal swabs, and the hospital environment itself, which acts as both a reservoir and vector for resistant organisms^40^. By assessing the resistome diversity and its microbial carriers across these interconnected domains, we aim to identify specific AMR determinants and the microbial taxa most frequently associated with their transmission.

Another distinguishing aspect of this study is the focus on the oropharyngeal microbiome, not just as a patient-related niche, but as a potential “entry point” for downstream infections and AMR spread. While the gut has long been studied as a reservoir of resistance, our inclusion of the oropharyngeal community as a critical hub for cross-compartmental transmission adds a novel perspective.

Importantly, we extend these analyses to patient outcomes, including mortality, enabling us to investigate the association between microbiome and resistome profiles and survival duration. By stratifying patients based on survival length, we aimed to identify microbiome-based biomarkers that could serve as early predictors of poor prognosis or heightened risk for adverse events. We also compared oropharyngeal and rectal swabs to assess which site better captures microbiome shifts relevant to disease progression and clinical deterioration.

Finally, our approach also has translational value. By pinpointing key resistances and their typical microbial vectors within ICU ecosystems, our findings could inform the development of more precise monitoring systems and antimicrobial strategies. By examining samples over time, we also aimed to track the emergence of novel resistance mechanisms under sustained antimicrobial pressure, contributing to a deeper understanding of resistance evolution in critical care. This is especially important in units where empirical antibiotic therapy remains unavoidable; a better understanding of ICU-specific resistance landscapes can support more effective, context-aware interventions that minimize the amplification and spread of resistant strains.

## Results

### Description of the study

**Fig. 1:**
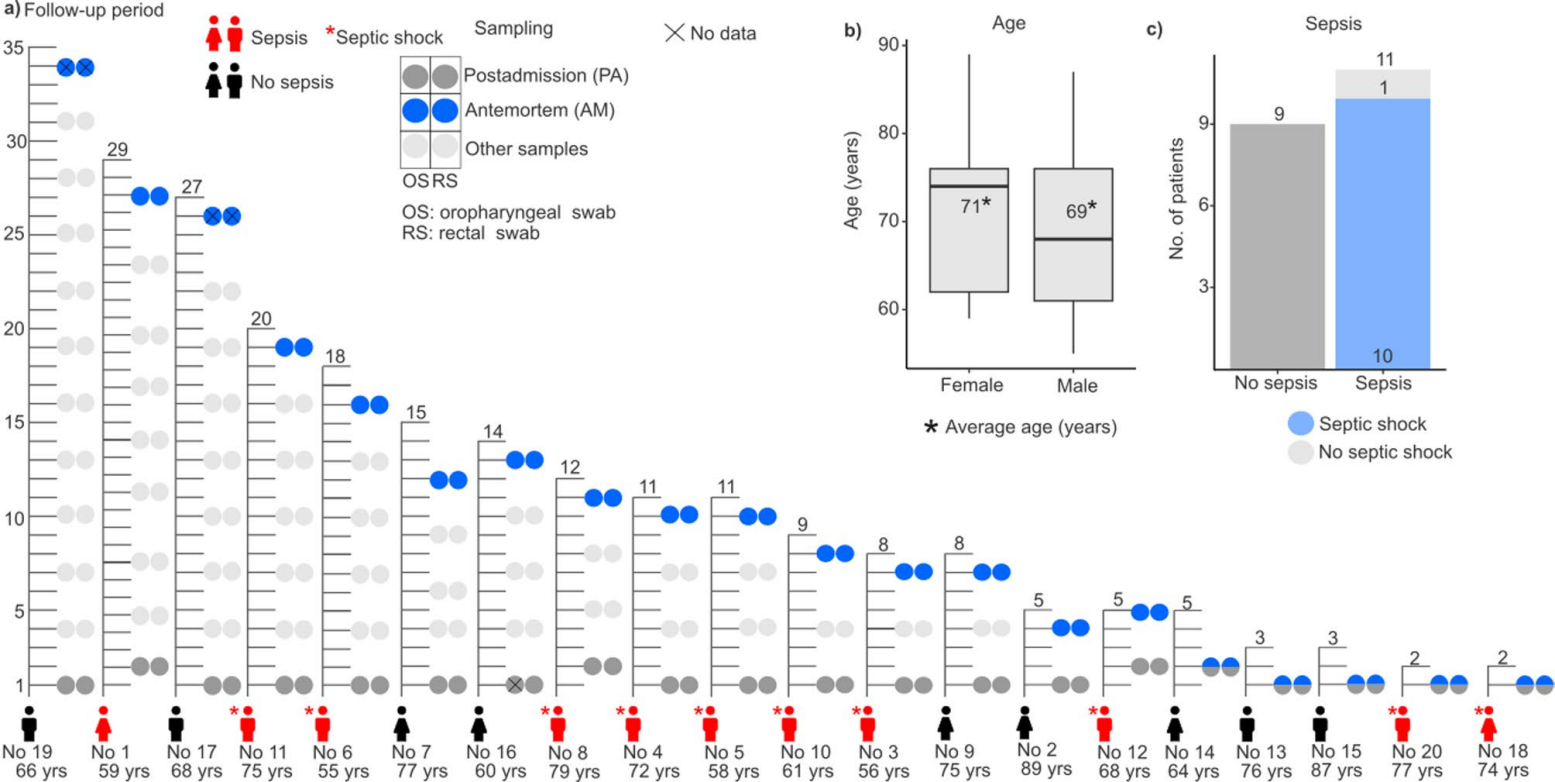
Overview of the study cohort. (a) The figure presents data from 20 patients included in the study, detailing their gender, age, presence of sepsis or septic shock, and ICU length of stay until death. It also illustrates the timeline of simultaneous oropharyngeal (OS) and rectal swab collections (RS), distinguishing between postadmission (PA) and antemortem (AM) samples to track changes throughout their ICU stay and near the time of death. (b) Boxplot illustrates the age of patients based on gender, with an asterisk indicating the mean age (years) of each group. (c) Bar plot shows the number of patients without and with sepsis.

Our follow-up study involved 69 intensive care unit patients admitted between February 13 and June 22, 2023, with oropharyngeal and rectal swab samples collected every three days. Inclusion criteria required adult patients who died in the ICU and had an anticipated stay of at least 48 h. From the 20 deceased patients (13 males, 7 females), with an average age of 69.8 ± 9.9 years (71 ± 10.8 years for female, and 69 ± 9.8 years for male patients) and a median hospital stay of 12.1 days (range: 2–35 days), samples were divided into two cohorts: postadmission and antemortem, with the latter collected on the day of their death or 1–2 days prior (Fig. 1a and b). Samples were collected from the Vas County Markusovszky University Teaching Hospital, Central Anesthesiology and Intensive Patient Care Department, Szombathely, Hungary. Treatment during hospitalization included mechanical ventilation for all deceased patients upon ICU admission. Medical histories revealed various pre-existing conditions, with 10 patients having cardiovascular, 9 patients having lung diseases, and 16 patients also suffering from hypertension. The majority of admissions (70%) were due to pulmonary issues like chronic obstructive pulmonary disease (COPD) and pneumonia. 11 patients (55%) were diagnosed with sepsis, leading to septic shock in 10 patients (Fig. 1c). Additionally, four medical workers - two nurses and two doctors (three males and one female) - participated in the study, collecting samples and providing continuous patient care throughout the follow-up period. Their average age was 48 ± 7.39 years. Moreover, environmental samples were obtained using specimen collection swabs from various surfaces such as bedrails, handwashing facilities, sinks, hospital spouts, taps, Astrup devices, Continuous Renal Replacement Therapy (CRRT) equipment, nurse’s stations, keyboards, telephones, airlock buttons, GE Healthcare ultrasound system, door handles, rapid tests (glucose, lactate), desks, storage rooms, laboratories, and patient rooms. In total, 23 environmental samples were collected (see **Supplementary File 1**). The patient cohort partially overlaps with our previously published study^35^; however, the current investigation expands upon it by including healthcare personnel and environmental sampling, and applies a distinct analytical framework focused on antimicrobial resistance dynamics.

### Antibiotic usage in the study group

In our study cohort, antibiotic treatments varied: monotherapy was administered to five patients (25%), dual therapy to ten patients (50%), four patients (20%) received a combination of monotherapy, dual therapy, and triple therapy, and there was one patient (5%) who received no antibiotic therapy during their hospitalization. Antibiotic administration was according to established guidelines^41^, and in every case, the recommended maximal dosages were ensured for optimal therapeutic efficacy.

### **Distribution of antimicrobial resistance and multidrug-resistant bacteria across ICU environments**,** patients**,** and healthcare staff**

**Fig. 2:**
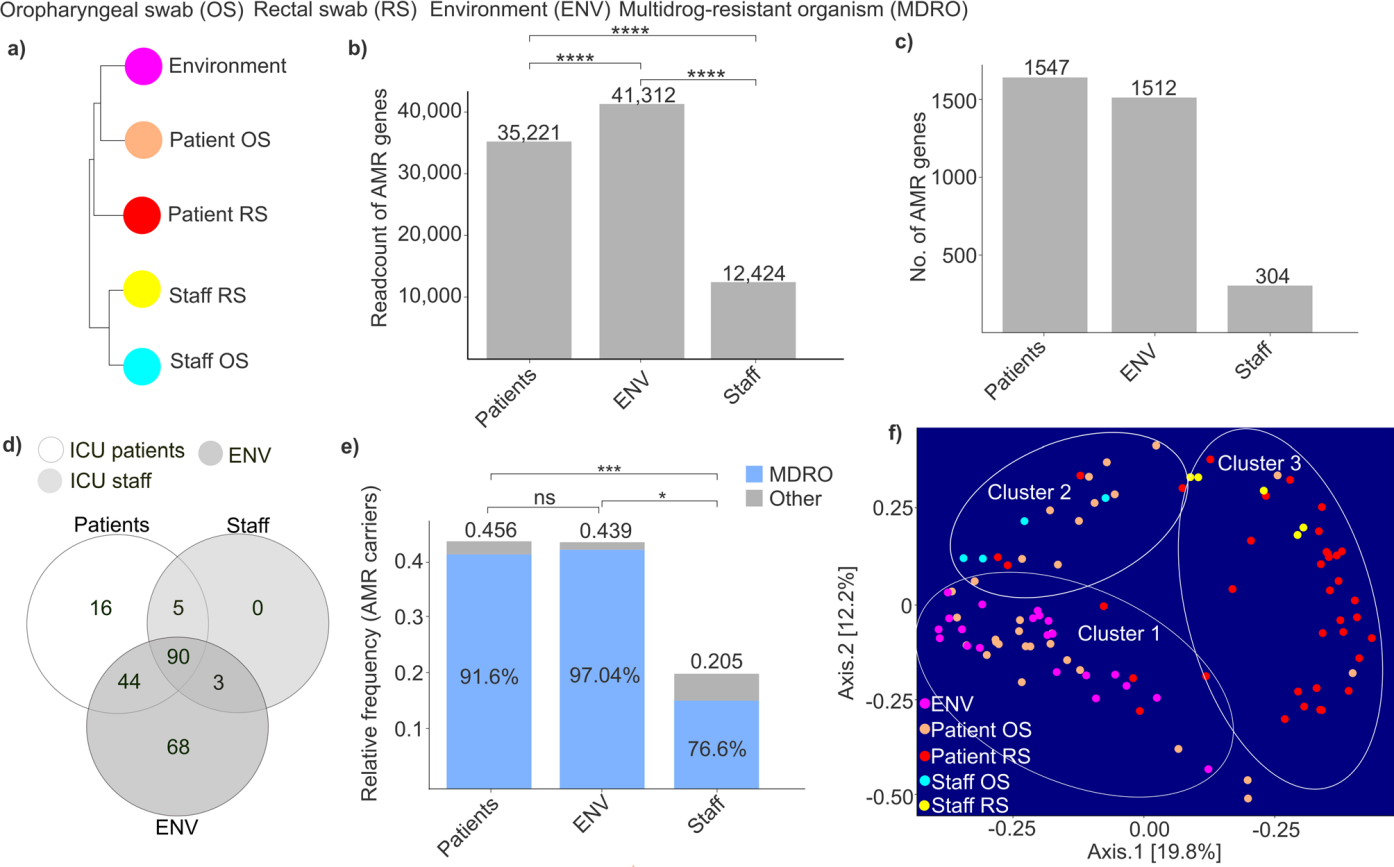
Illustration of the interrelation between taxonomy and resistome data of microbiota from various sources. (**a**) Cluster analysis shows how different sample types relate to each other. Sample types: environment (ENV), patient’s oropharyngeal (OS), and rectal swab (RS), and medical staff’s oropharyngeal, and rectal swab samples. (**b**) Bar plots represent the read count of antimicrobial resistance (AMR) genes in different groups: patients, environment, and medical staff. (**c**) Bar plots show the number of AMR genes in different groups: patients, environment, and medical staff. (**d**) A Venn diagram estimates the variety of shared and unique AMR-associated pathogens among healthcare staff, critically ill patients, and the ICU environment. (**e**) Bar plots represent the relative frequency of AMR carriers in different groups: patients, environment, and medical staff. (f) PCoA plots were created to investigate potential clustering pattern similarities based on data of multidrug-resistant organisms (MDROs), between different sample types, described previously. Each point is a single sample and the distance between them is proportional to the difference. Asterisks indicate significant differences (*p < 0.05, ***p < 0.001, ****p < 0.0001).

A cluster analysis was conducted to clarify the codependent patterns in antimicrobial resistance across microbiota from various sources of the hospital environment in the ICU, as well as those of healthcare professionals and critically ill patients, with a special focus on different anatomical sites, such as the oropharynx and rectum (Fig. 2a).

Our analysis revealed that the environment had the most distinct composition of AMRs, yet it also showed the most similarity to patient microbiota, particularly to oropharyngeal swabs (OS), followed by rectal swabs (RS).

We assessed how the read count of AMR genes varied across our experimental groups (Fig. 2b). Our findings revealed that the highest read counts were observed in ICU environmental samples, which were significantly higher compared to both patient and staff samples (Wilcoxon rank-sum test, Environment AMR read count: 41,312 vs. Patient AMR read count: 35,221, p-value < 0.0001; Environment AMR read count: 41,312 vs. Staff AMR read count: 12,424, p-value < 0.0001). Additionally, we measured significantly lower read counts in staff samples compared to ICU patient samples (Wilcoxon rank-sum test, Patient AMR read count: 35,221 vs. Staff AMR read count: 12,424, p-value < 0.0001).

When examining the number of different AMR gene types across our groups, we found that staff samples contained the lowest number of AMR variants. ICU patient samples contained 5.08 times higher and environmental samples 4.97 times higher number of AMRs compared to staff samples (Fig. 2c). Meanwhile, patients exhibited higher AMR diversity than environmental samples (Number of AMR genes, patients: 1,547 vs. environment: 1,512).

Another objective was to compare the diversity of antibiotic-resistant organisms (AROs) present in patients, healthcare staff, and the ICU environment, as well as to assess the extent of overlap between these different habitats (Fig. 2d). A Venn diagram was used to identify shared and unique AROs. Among the detected organisms, an exceptionally high proportion (90.71%) of resistance-carrying microbes, based on the CARD database, were found in the environment, with 68 species (30% of the total) being unique. This underscores the ICU environment as a significant reservoir of diverse AMRs. Patient samples contained 68.58% of the identified AROs, with 7% being unique to this group. In contrast, staff samples harbored only 43.36% of the AROs (98 species), none of which were unique. Notably, 90 species, representing 40% of the total detected AROs, were shared across all sample groups. When analyzing the common AROs, the genera *Streptomyces* and *Streptococcus* exhibited notably high abundance (*Streptomyces*: 8 species, *Streptococcus*: 7 species). At the phylum level, *Firmicutes*, *Proteobacteria*, and *Actinobacteria* dominated the resistome composition, collectively accounting for approximately 69% of all identified shared AROs (*Firmicutes*: 27 species, *Proteobacteria*: 19 species, and *Actinobacteria*: 16 species).

Examining the relative prevalence of AMR-carrying species, we found that the highest proportion of AROs was observed in patient samples (Fig. 2e). Although a lower prevalence of AROs was measured in the environment, the difference was not statistically significant (Wilcoxon rank-sum, Patients ARO relative frequency: 0.456 vs. environment ARO relative frequency: 0.439, p-value = 0.198). The lowest proportion of AROs was detected in staff samples, which was significantly lower compared to both patient and environmental samples (Wilcoxon rank-sum, staff ARO relative frequency: 0.205 vs. patient ARO relative frequency: 0.456, p-value = 0.0008; staff ARO relative frequency: 0.205 vs. environmental ARO relative frequency: 0.439, p-value = 0.0104). Additionally, we assessed the proportion of AROs classified as multidrug-resistant organisms. Our findings showed that the highest percentage was observed in environmental samples (97.04%), while the lowest was detected in staff samples (76.6%).

The coherence between the assortment of MDROs in various sample groups was then analyzed (Fig. 2f). This investigation examined the distribution and diversity of MDROs across environmental sources and the microbiota of patients and staff, using oropharyngeal and rectal swab samples. The PCoA plot revealed three distinct clusters based on MDRO data: ‘Cluster 1’ included samples from both the environment and patients’ oropharyngeal swabs; ‘Cluster 2’ showed an overlap between staff oropharyngeal, patient oropharyngeal, and patient rectal swabs; and ‘Cluster 3’ displayed overlap between patient oropharyngeal, patient rectal, and staff rectal samples. Patients’ oropharyngeal swabs showed notable MDRO overlap with environmental sources and, to a lesser extent, with staff oropharyngeal samples. Interestingly, while the overlap with environmental samples was substantial, the similarity with staff microbiota, especially rectal samples, was comparatively less noteworthy. Additionally, neither patient nor staff rectal samples showed substantial overlap with environmental MDROs.

### Distribution of AMRs associated with healthcare-associated infection-causing pathogens in various resistance reservoirs

**Fig. 3:**
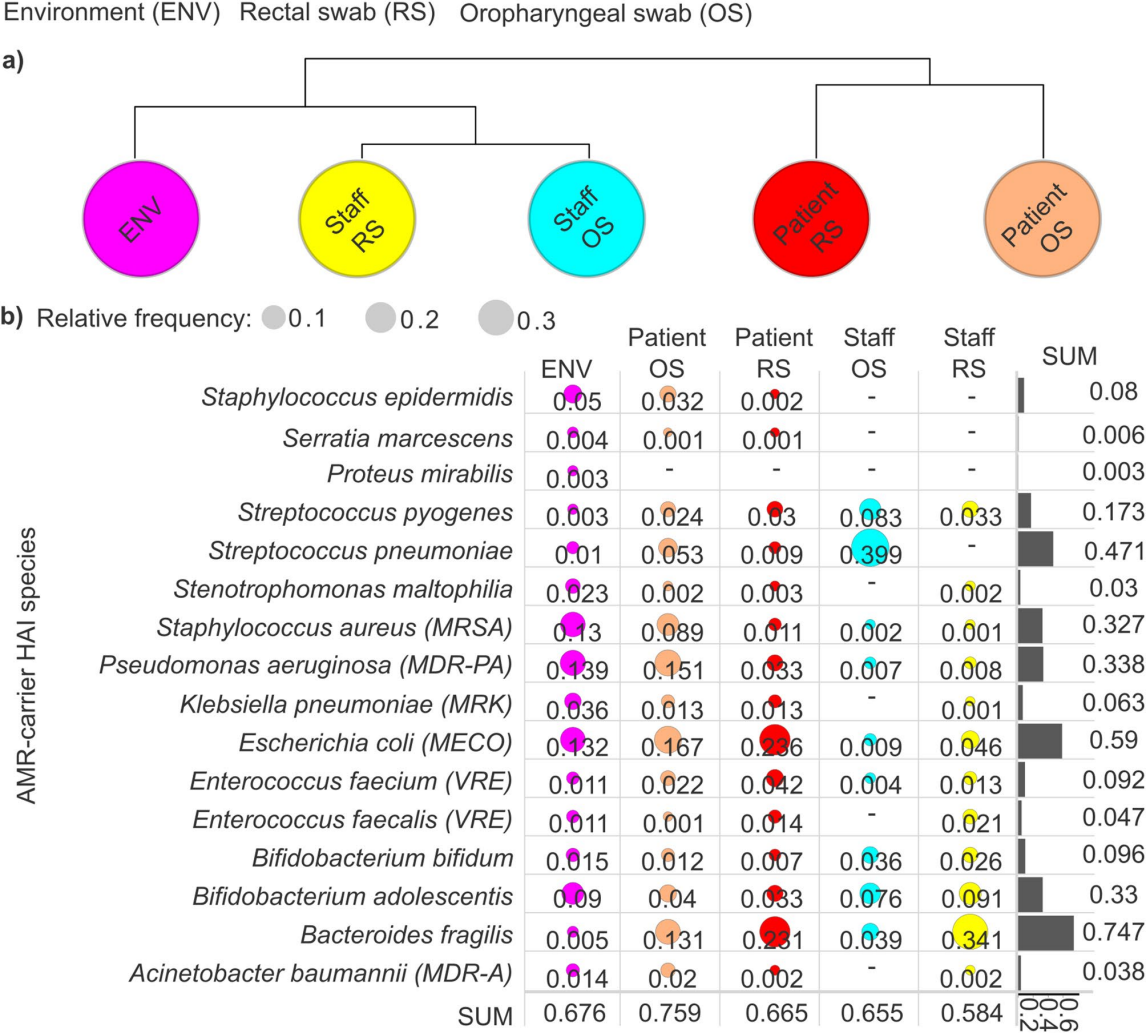
Distribution and prevalence of antimicrobial resistances (AMRs) associated with healthcare-associated infection (HAI)-causing species across sample groups. (**a**) A cluster map was generated to visualize the relationships between sample groups based on HAI AMR relative abundance data, revealing interconnected patterns among healthcare staff oropharyngeal swabs (staff OS), healthcare staff rectal swabs (staff RS), patient oropharyngeal swabs (patient OS), patient rectal swabs (patient RS), and environmental samples (ENV). (**b**) Bubble plots display the prevalence of AMR associated with HAI species across sample groups, with bubble diameter proportional to their relative abundance. Bar plots show the cumulative relative frequency of each species across the groups.

Cluster analysis revealed distinct structural patterns in AMR assortments associated with healthcare-associated infection-causing species (Fig. 3a). AMR profiles were most similar among patient-derived oropharyngeal and rectal swabs, while those from asymptomatic healthcare personnel also exhibited strong internal similarity. In contrast, environmental samples showed the greatest divergence from HAI-associated AMRs. Notably, staff-associated AMR profiles resembled greater similarity to environmental samples than to those derived from patients.

We examined the distribution of AMR determinants carried by HAIs-causing species across different sample types, including environmental, patient (oropharyngeal and rectal swabs), and staff microbiome samples (Fig. 3b). Our analysis revealed that HAI-associated AMR occurrence was highest in patient oropharyngeal swabs (0.759), indicating a significant reservoir within this anatomical niche. Environmental samples followed with a cumulative AMR frequency of 0.676, suggesting substantial contamination and potential transmission pathways. Patient rectal swabs exhibited comparable AMR frequencies (0.665), followed by staff oropharyngeal swabs (0.655), reinforcing the hypothesis that asymptomatic healthcare personnel could serve as significant carriers. Notably, the lowest cumulative AMR frequencies were observed in rectal swabs from healthcare staff (0.584).

Across the examined HAI niches, *Bacteroides fragilis* exhibited the highest AMR association (rf: 0.747), followed by *Escherichia coli* (rf: 0.59), *Streptococcus pneumoniae* (rf: 0.471%), *Pseudomonas aeruginosa* (rf: 0.338), and *Staphylococcus aureus* (rf: 0.327). Notably, our data revealed that *B. fragilis*-associated AMRs were most prevalent in staff rectal swabs (rf: 0.341), followed by patient rectal swabs (rf: 0.231) and patient oropharyngeal swabs (rf: 0.131). In contrast, *E. coli* exhibited the highest AMR frequencies in patient rectal swabs (rf: 0.236), with slightly lower but still considerable prevalence in patient oropharyngeal swabs (rf: 0.167) and environmental samples (rf: 0.132). *S. pneumoniae* displayed an exceptionally high AMR prevalence in staff oropharyngeal swabs (0.399), while its presence in other niches was markedly lower. In staff rectal swabs, no *S. pneumoniae*-associated AMR determinants were detected. Among HAI-associated species, *E. coli* emerged as the dominant AMR carrier in patient oropharyngeal swabs (rf: 0.167), followed by *P. aeruginosa* (rf: 0.151), *B. fragilis* (rf: 0.131), and *S. aureus* (rf: 0.083). In contrast, *S. pneumoniae* was the predominant AMR-carrying species in staff oropharyngeal swabs.

### Identification and comparative distribution of common antimicrobial resistances (CAMRs) across various ICU-associated niches

**Fig. 4:**
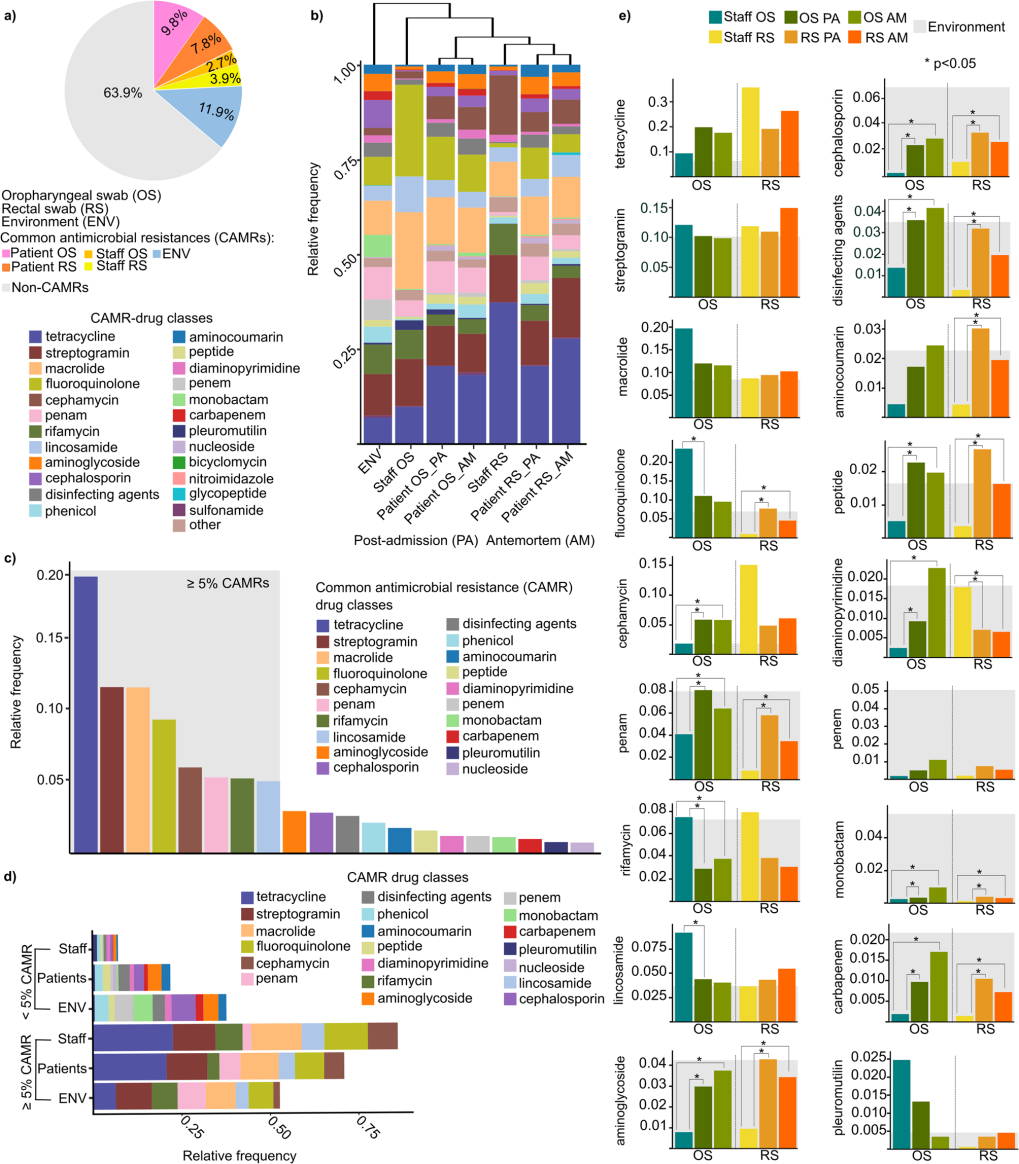
Distribution of common antimicrobial resistances (CAMRs). **(a)** A pie chart demonstrating the distribution of non-CAMRs and CAMRs in patients’ oropharyngeal and rectal swabs, as well as in the staff’s oral and rectal swabs and in the environment. **(b)** Clustered stacked bar plot shows patterns and similarities in CAMRs across sample types. **(c)** Bar plot shows the relative frequency of CAMRs with a relative frequency above and below 5% relative frequency in our study cohort. **(d)** Stacked bar plots show the distribution of CAMR classes relative frequency across the different sample populations. **(e)** Differences in the relative distribution of CAMRs associated with staff and patients in both oropharyngeal (OS) and rectal swab (RS) samples were examined, as well as to postadmission (PA) and antemortem (AM) samples. The CAMRs load of the environment were marked with light grey background. In every case, bar plots were used to examine the statistical differences between the sample groups. Asterisks indicate significant difference (*p < 0.05).

Common antimicrobial resistances (CAMRs) displaying an above-average relative occurrence were identified (see **Supplementary File 2**). The CAMRs accounted for 36.1% of all detected resistances (Fig. 4a). CAMRs showed the highest frequency in environmental samples (11.9%), followed by patient samples, where oral swabs exhibited the highest frequencies (9.8%), surpassing the values measured in patients’ rectal swabs (7.8%). The lowest distribution of CAMRs was observed in staff samples, with the lowest values found in their OS samples (2.5%), while slightly higher values were recorded in their RS samples (3.9%).

Resistance classes associated with the CAMRs were also analyzed (Fig. 4b). Hierarchical clustering was applied to uncover patterns in the respective resistance classes across different sample groups, providing a visual representation of the distribution patterns. The results revealed that environmental samples differed the most from swabs collected from the anatomical sites, such as the oropharynx and rectum. It was estimated that for the CAMRs, the OS samples, particularly those from the healthcare staff, showed the greatest resemblance to the environmental samples. Interestingly, for both anatomical sites, the postadmission (PA) patterns were more similar to the environmental samples than the antemortem (AM) ones. However, a Kruskal–Wallis test did not reveal statistically significant differences in resistance class distributions across the sample groups (χ² = 5.69, *p* = 0.46).

A distribution diagram was created to display resistance classes by their relative occurrences, highlighting CAMRs with relative frequencies below and above 5% (Fig. 4c). Based on these, tetracycline was the most abundant (rf: 20.7 ± 0.1%) from CAMR-drug classes in our sample cohort, followed by streptogramin and macrolide (streptogramin rf: 12.4 ± 0.02%, macrolide rf: 12.4 ± 0.04%), fluoroquinolone (rf: 10 ± 0.07%), cephamycin (rf: 6.4 ± 0.04%), penam (rf: 5.7 ± 0.03%), rifamycin (rf: 5.6 ± 0.02%), and lastly, lincosamide (rf: 5.4 ± 0.02%). A Kruskal–Wallis test revealed significant differences in the relative frequencies across resistance classes (χ² = 98.57, *p* < 0.0001), indicating notable variation in the distribution of resistance across the different classes.

When examining CAMRs with frequencies below 5%, contrasting abundance patterns were observed compared to previous findings. The environment showed the highest burden for CAMRs below 5%, while staff samples had the lowest. Conversely, CAMRs with frequencies above 5% were primarily found in staff samples, followed by patient samples, and were least common in environmental samples (Fig. 4d). A Kruskal–Wallis test confirmed that these differences in relative frequencies across sample groups were statistically significant (χ² = 46.16, *p* < 0.0001).

The magnitude of the differences in the relative frequencies of CAMRs was also calculated statistically by using Wilcoxon rank-sum test (Fig. 4e). Upon analyzing resistance class frequencies, tetracycline had the lowest environmental burden (Environment rf: 0.063) compared to patients and staff (rf: 0.21 ± 0.089), followed by cephamycin (Environment rf: 0.018, others: 0.066 ± 0.045), pleuromutilin (Environment rf: 0.004, others: 0.008 ± 0.0091), and fluoroquinolone (Environment rf: 0.070, others: 0.096 ± 0.077). Despite its low environmental burden, tetracycline showed the highest relative frequencies in patient and staff samples, particularly in staff rectal swabs (rf: 0.35) and patients’ dysbiotic antemortem samples (rf: 0.26). Some resistance classes, such as carbapenem and aminoglycoside, showed significantly lower burdens in staff OS and RS samples despite high environmental loads (carbapenem Staff OS rf: 0.0019 vs. carbapenem OS PA rf: 0.0097, OS AM rf: 0.017, *p* < 0.05; carbapenem Staff RS rf: 0.0014 vs. carbapenem RS PA rf: 0.010, RS AM rf: 0.0072, *p* < 0.05; aminoglycoside Staff OS rf: 0.0079 vs. aminoglycoside OS PA rf: 0.030, OS AM rf: 0.037, *p* < 0.05; aminoglycoside Staff RS rf: 0.0095 vs. aminoglycoside RS PA rf: 0.043, RS AM rf: 0.034, *p* < 0.05). Among the CAMRs with a frequency greater than 5% relative frequency, we observed lower rifamycin resistance values in both the patients’ OS and RS samples compared to the staff samples, despite the high environmental burden. Additionally, these differences were found to be statistically significant (OS patient PA rf: 0.029, OS AM rf: 0.038 vs. OS staff rf: 0.075, *p* < 0.05) in the OS samples.

### Investigation of the extent of microbial translocation and AMR dynamics in early versus late mortality ICU patients

**Fig. 5:**
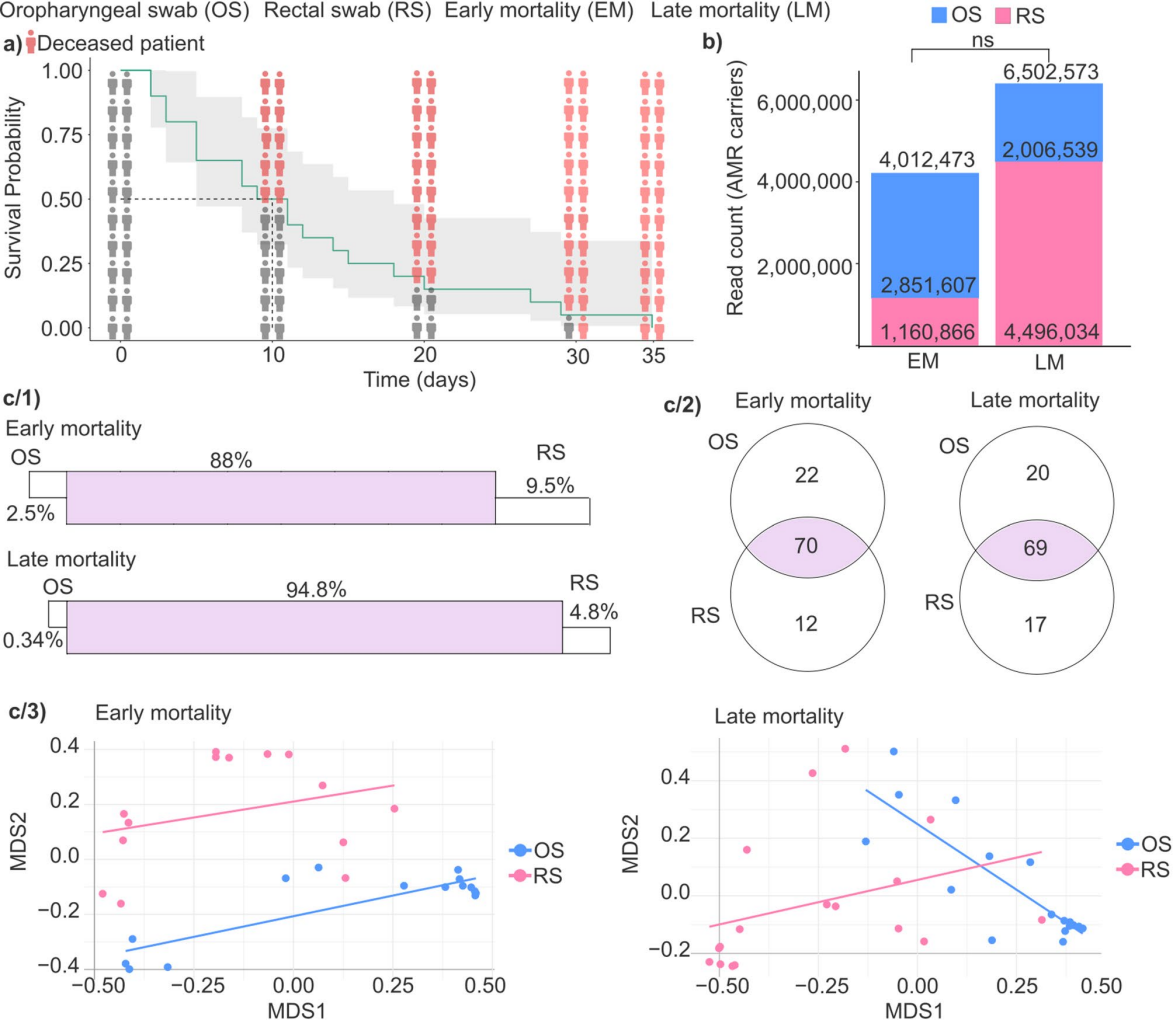
Analysis of survival and antimicrobial resistance in ICU patients. (**a**) The Kaplan-Meier curve was used to analyze the patient’s death time in our study population. The y-axis shows the survival probability, x-axis the time spent on the ward. The icons show the survival probability on the 0, 10, 20, 30 days. Red icons illustrate the deceased patients. (**b**) Stacked bar plots represent the antimicrobial resistance (AMR) read count in early mortality (EM) and late mortality (LM) groups. Color represents different anatomical sites (blue OS = oropharyngeal swab, pink RS = rectal swab).  (c/1) Panels display the degree of overlap in AMR carrier bacterial taxa between anatomical sites in both early and late mortality groups. (c/2) Venn diagrams illustrate the distribution of AMR carrier bacterial taxa between OS and RS samples within the early and late mortality groups. (c/3) Multidimensional scaling (MDS) plots with linear regression lines were used to visualize the composition of core AMR carrier species in OS and RS samples. NS = not significant.

The Kaplan–Meier estimator was utilized to evaluate patient survival, illustrating survival probabilities across a 0-35-day follow-up period, stratified by patients’ length of hospital stay prior to death (Fig. 5a). The median survival time, defined as the time at which 50% of patients had died, was 10 days (28.6% of the total study duration). Based on this threshold, the patient population was stratified into two groups: those who experienced early mortality (EM), defined as death within 10 days, and those who experienced late mortality (LM), defined as survival beyond 10 days.

Subsequently, we determined the read counts of AMR carriers in patient samples from two anatomical regions, the oropharynx and the rectum, to compare the EM and LM groups (Fig. 5b). The cumulative number of AMR read counts was 4,012,473 in the EM group and 6,502,573 in the LM group, representing a 1.62-fold increase in LM patients (Wilcoxon rank-sum test, not significant, p-value = 0.987). Within the OS group, AMR read counts were higher in EM patients, showing a 1.42-fold increase compared to LM patients (Wilcoxon rank-sum test, EM = 2,851,607 vs. LM = 2,006,539, not significant, p-value = 0.496). Conversely, in the RS group, AMR read counts were 3.87 times higher in LM patients than in EM patients (Wilcoxon rank-sum test, EM = 1,160,866 vs. LM = 4,496,034, not significant, p-value = 0.883), also without statistical significance.

The **microbial translocation** of AMR-carrying species between anatomically distant microbiome sites was investigated by comparing oropharyngeal and rectal swab samples in both the EM and LM patient groups (Fig. 5c). Our findings indicate that the level of bacterial translocation of AMR carrier species was higher in the LM group compared to the EM group (Fig. 5c**/1**). Specifically, the prevalence of core AMR-carrier species - defined as species detected in both anatomical sites - was higher in the LM group (94.8%) compared to the EM group (88%), suggesting increased microbial exchange between anatomical locations among patients with prolonged survival. Additionally, in both patient groups, rectal swab samples consistently showed a higher proportion of unique species compared to oropharyngeal swab samples. Specifically, in the EM group, unique species were 3.8-fold higher in RS (9.5%) than OS (2.5%), while in the LM group, this difference was even more pronounced, with RS (4.8%) showing 14.1-fold more unique species than OS (0.34%).

Venn diagrams were made to illustrate the variety and the distribution of AMR carrier species between OS vs. RS across the early-mortality and late-mortality patient groups (Fig. 5c**/2**). In the EM group, 21.2% of the detected species (22 out of 104) were unique to OS samples, while 11.5% (12 out of 104) were exclusive to RS samples, whereas the majority (67.3%, 70 out of 104) of identified species were shared between both anatomical sites, constituting the core AMR carrier microbiome. In the LM group, the proportion of OS-specific species was slightly lower (18.9%, 20 out of 106) compared to the EM group, whereas the proportion of RS-specific species increased to 16.0% (17 out of 106). The proportion of core species common to both OS and RS samples remained similar to that observed in the EM group, at 65.1% (69 out of 106).

Linear regression lines overlaid on MDS plots illustrate the relative compositional relationship between AMR-carrying core microbiota in oropharyngeal and rectal swab samples, highlighting patterns of similarity or divergence in microbial community structure across these anatomically distinct sites (Fig. 5c**/3**). In the early mortality group, we observed a modest but significant alignment between oropharyngeal and rectal microbiomes along the first MDS axis (MDS1 coefficient = 0.30, *p* = 0.0099), suggesting a degree of coordination or similarity in AMR carrier distribution across these anatomical sites. Rectal swabs displayed a slight positive trend, potentially indicating that changes in AMR abundance in one site were mirrored in the other, pointing to interdependence between the two microbial communities.

In contrast, the late mortality group exhibited a more complex pattern: oropharyngeal samples showed a strong negative trend along MDS1 (coefficient = −0.91, *p* = 0.0005), while rectal samples maintained a positive trajectory. A significant interaction term (MDS1:GroupRS = 1.22, *p* = 0.0003) further supports increased convergence between OS and RS microbiota over time, possibly reflecting enhanced microbial exchange or stabilization of AMR-carrying populations in patients with prolonged ICU stays.

### Identification of microbial biomarkers predictive of severe clinical deterioration and early mortality in ICU patients

Although our study cohort exclusively comprised ICU-admitted patients who died during hospitalization, one of our primary objectives was to distinguish patients who experienced early mortality (EM) from those with comparatively longer survival (LM), based on a median survival threshold. Accordingly, we aimed to identify microbial taxa in postadmission oropharyngeal and rectal swab samples that may serve as potential biomarkers of severe dysbiosis, possibly associated with imminent clinical deterioration shortly after ICU admission.

**Fig. 6:**
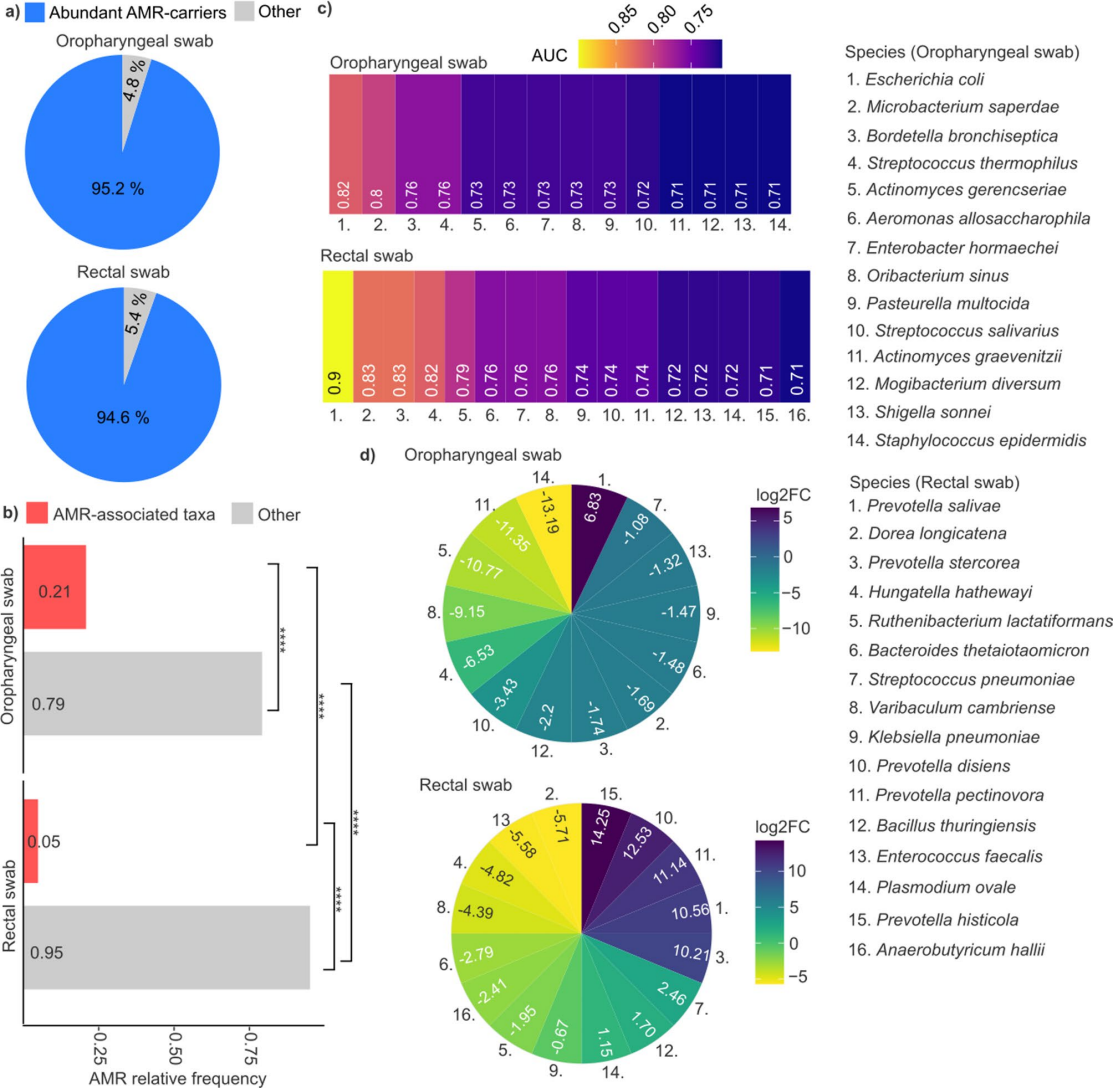
Identification of microbial biomarkers associated with mortality. (**a**) Pie charts visualize AMR-carrier taxa relative frequency > 0.001 (blue) and relative frequency < 0.001 (grey). (**b**) Bar plots represent relative frequency of oropharyngeal (OS), and rectal swab (RS) sample. Red bars mean AMR-associated taxa, while grey means non-AMR-associated. (**c**) Heatmaps visualize Area under curve (AUC) values of oropharyngeal swab, and rectal swab samples. (**d**) Circular heatmaps represent the logarithm of fold change (log2FC) in oropharyngeal swab, and rectal swab samples. Positive values (blue color) mean more abundant in early mortality samples, and negative values (yellow color) mean more abundant in late mortality samples. Species are listed on the right side, for both oropharyngeal and rectal swab samples. Asterisks indicate significant differences (**** *p* < 0.0001).

To reliably identify microbial biomarkers predictive of clinical outcomes, our analysis specifically focused on taxa exhibiting relatively high abundance - defined as abundant AMR-carrying microbes with relative abundance > 0.001 (abundant AMR-carriers). Collectively, these taxa accounted for approximately 95% of all detected species in both oropharyngeal swab (OS; relative frequency: 0.952 ± 0.033) and rectal swab (RS; relative frequency: 0.946 ± 0.028) samples (Fig. 6a).

Receiver operating characteristic (ROC) curves were generated to evaluate the discriminative power of abundant AMR-carrier biomarkers in distinguishing ICU-admitted patients with early mortality (survival < 10 days, *n* = 10) from those with late mortality (survival up to 35 days, *n* = 10). To achieve this, relative abundance data from postadmission swab samples were converted into binary variables reflecting prospective clinical outcomes (EM vs. LM), and the area under the curve (AUC) was calculated to identify microbial taxa with the highest prognostic potential (**Supplementary File 3**).

Subsequently, taxa with high relative abundance (rf > 0.001) and strong discriminatory power (AUC > 0.7) were identified. The relative frequency of AMR-associated taxa was significantly higher in OS compared to RS samples, representing a 4.2-fold difference (Wilcoxon rank-sum test, OS rf: 0.21 vs. RS rf: 0.05, *p* < 0.0001) (Fig. 6b). Fourteen such taxa were detected in OS samples, whereas sixteen were found in RS samples (Fig. 6c). Notably, taxa from RS samples exhibited generally stronger discriminatory power, including *Prevotella salivae* (AUC = 0.9), *Dorea longicatena* (AUC = 0.83), *Prevotella stercorea* (AUC = 0.83), and *Hungatella hathewayi* (AUC = 0.82). Overall, the mean AUC of the top four microbial biomarkers was higher in RS (0.85 ± 0.04) than in OS samples (0.78 ± 0.03).

Subsequently, taxa exhibiting significant differences in relative abundance between EM and LM patients were identified by calculating the log₂ fold-change (EM/LM) based on postadmission swab samples (Fig. 6d). In oropharyngeal samples, *Escherichia coli* showed the highest increase (log₂ fold-change: 6.82) in EM compared to LM patients. In rectal swabs, the most pronounced increases in EM patients were observed for *Prevotella histicola* (log₂FC = 14.25), *P. disiens* (log₂FC = 12.53), *P. pectinovora* (log₂FC = 11.14), *P. salivae* (log₂FC = 10.56), and *P. stercorea* (log₂FC = 10.56). In contrast, the greatest decreases occurred in *Dorea longicatena* (log₂FC=−4.82), *Hungatella hathewayi* (log₂FC=−4.82), *Varibaculum cambriense* (log₂FC=−4.39), and *Enterococcus faecalis* (log₂FC=−5.58) in EM relative to LM patients.

### Identification of multidrug-resistant organisms as early biomarkers for sepsis in ICU patients

**Fig. 7:**
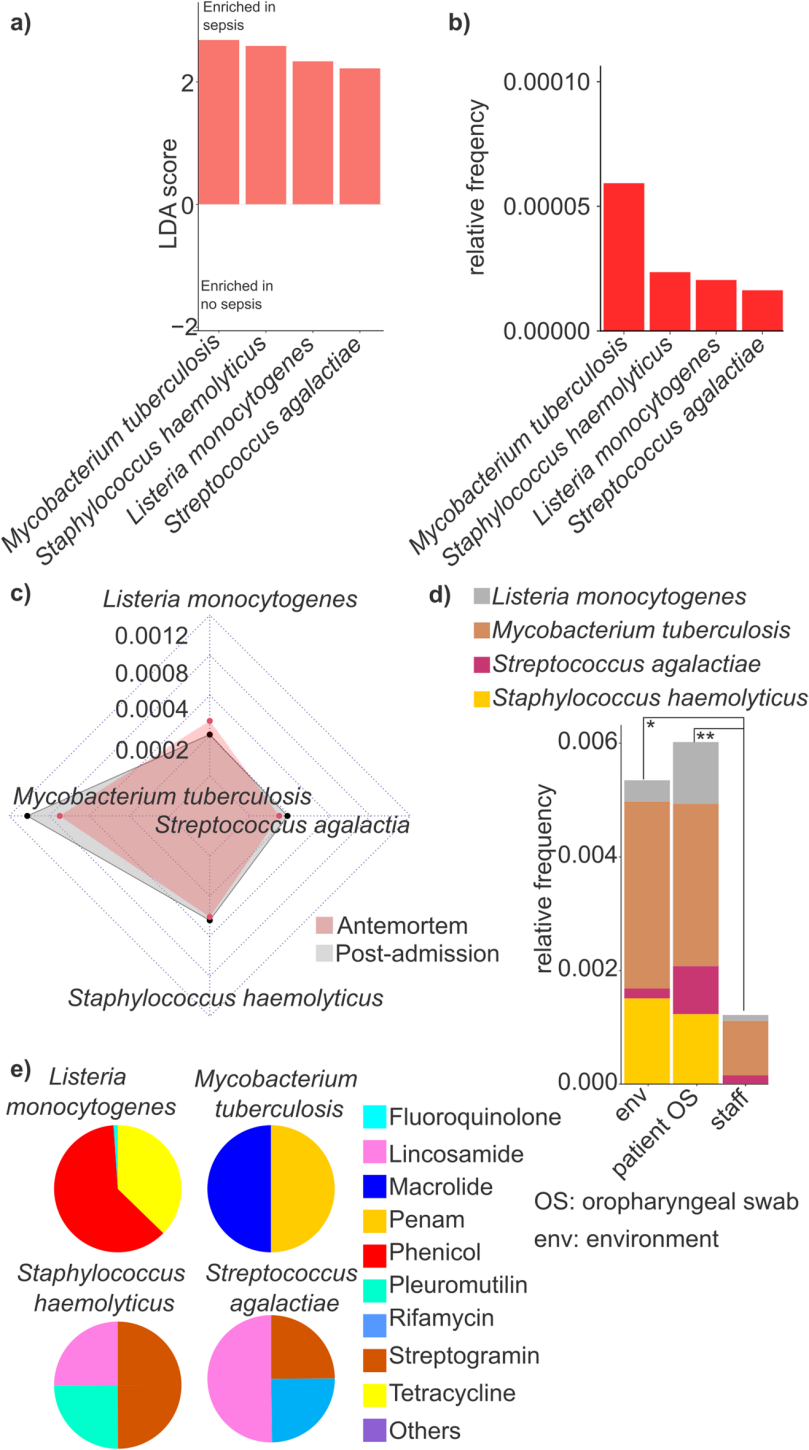
Comparison of taxonomic differences and antimicrobial associations in sepsis and non-sepsis samples. (**a**) Bar plots represent the Linear discriminant analysis (LDA) score > 2 values taxons between sepsis and no sepsis samples. (**b**) The relative frequencies of these bacteria were visualized with bar plots. (**c**) Radar chart was used to estimate the differences between postadmission and antemortem samples regarding the selected taxons. (**d**) The distribution of the selected taxons was checked in different sample types. (**e**) Pie charts were used to visualize the 9 antimicrobial classes that related to the selected taxons. Asterisks indicate significant difference (* *p* < 0.05, **p < 0.01).

In this study, we also aimed to identify MDROs in postadmission samples from ICU patients as potential early biomarkers predictive of sepsis development. For these Linear Discriminant Analysis Effect Size (LEfSe) was applied to identify microbial taxa significantly enriched in PA samples from patients who subsequently developed sepsis (10 patients, comprising 50% of the cohort, who ultimately succumbed to septic shock) compared to those who remained sepsis-free (10 patients, 50% of the cohort). Although our analyses included both oropharyngeal swab and rectal swab samples, only OS samples yielded taxa with an LDA score greater than 2 (Fig. 7a). Accordingly, four multidrug-resistant organism species - *Listeria monocytogenes*, *Mycobacterium tuberculosis*, *Staphylococcus haemolyticus*, and *Streptococcus agalactiae* - were found to be significantly enriched in postadmission samples of septic patients.

The relative abundance of these species was quantified, revealing that *M. tuberculosis* exhibited, on average, a 2.61-fold, significantly higher prevalence compared to the other species (Wilcoxon rank-sum test, *M. tuberculosis* rf: 0.000055 ± 0.000021 occurrence vs. the average of others: 0.000017 ± 0.000013; p-value = 0.0044) (Fig. 7b).

Next, the differences in the presence of these species between PA and AM samples were examined, focusing solely on OS samples (Fig. 7c). Interestingly, no significant differences were observed between PA and AM samples for *L. monocytogenes* (Wilcoxon rank-sum test, AM rf: 0.00041, PA rf: 0.00031, *p* > 0.05), *S. agalactiae* (Wilcoxon rank-sum test, AM rf: 0.00022, PA rf: 0.00028, *p* > 0.05), and *S. haemolyticus* (Wilcoxon rank-sum test, AM rf: 0.00045, PA rf: 0.00048, *p* > 0.05). However, a slightly elevated relative abundance of *M. tuberculosis* was detected in PA samples (AM rf: 0.00082, PA rf: 0.0011), although these differences were not statistically significant (Wilcoxon rank-sum test, *p* > 0.05).

The relative abundance of these species across different sample populations was further analyzed, revealing that the cumulative relative abundance of the four sepsis-enriched MDRO species was highest in OS samples from patients (rf sum: 0.0060, mean: 0.0015 ± 0.00091, p-value = 0.009), followed by the ICU environment (rf sum: 0.0052, mean: 0.0013 ± 0.0014, p-value = 0.02), in comparison with staff samples (rf OS combined: 0.0060 vs. staff) (Fig. 7d).

Finally, the resistance classes associated with sepsis-specific species were examined (Fig. 7e). Notably, considerable variation was observed in the distribution of resistance classes. Nine major resistance classes were identified, with *M. tuberculosis* exhibiting the highest number of associated AMR determinants, which were categorized into only two major classes: macrolides (50.0%) and penams (50.0%). Similarly, *L. monocytogenes* was linked to two main resistance classes, with phenicols being overwhelmingly dominant (61.6%), followed by tetracyclines (37.3%). In contrast, *S. haemolyticus* and *S. agalactiae* were associated with three major resistance classes, respectively, with lincosamides and streptogramin being common to both (*S. haemolyticus*: lincosamide 25.0%, pleuromutilin 25.0%, streptogramin, 50.0%; *S. agalactiae*: lincosamide: 50.0%, rifamycin, streptogramin each 25.0%).

### Patterns of antibiotic use and their association with newly emerged resistances

**Fig. 8:**
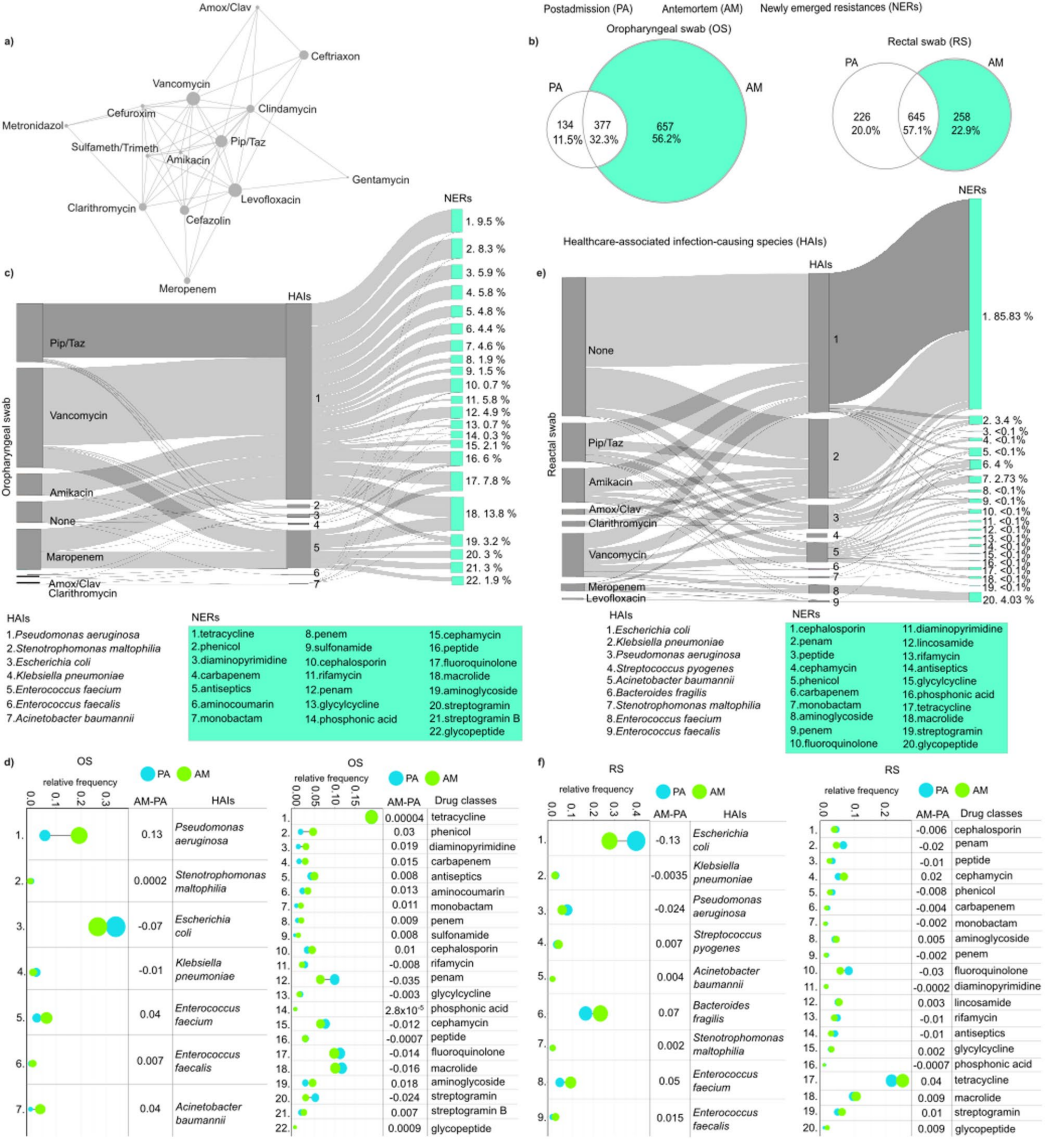
Antibiotic combination regimes and the development of newly emerged resistances (NERs). (**a**) A network illustrating the antibiotics, and their combinations utilized throughout the study period. Nodes symbolize antibiotics, with their size proportional to the extent of their use, while edges indicate their use in combination therapies. (**b**) Venn diagrams illustrating the number of newly acquired resistances in patients’ antemortem (AM) samples from oropharyngeal swabs (OS) and rectal swabs (RS). Sankey diagrams showing newly emerged resistance classes and their association with healthcare-associated infection (HAI)-causing species in patients’ (**c**) oropharyngeal and (**e**) rectal swabs. Relative frequency data showing the average distribution of NER-carrying species and the carried drug classes throughout our entire population in both our (**d**) OS and (**f**) RS sample groups.

A network diagram was designed to illustrate the antibiotics, and their combinations used throughout the follow-up period (Fig. 8a). The most frequently used antibiotics included vancomycin, levofloxacin, and piperacillin/tazobactam, often administered as part of combination therapies. In contrast, gentamicin and metronidazole were among the least commonly used antibiotics.

The occurrence of newly emerged resistances (NERs) in antemortem oropharyngeal and rectal swabs was also investigated. Based on in silico data, Venn diagrams revealed that a substantial proportion (56.25%) of the different types of resistances identified in microbiomes from oropharyngeal swabs were newly emerged indicating that these resistances were only detectable in the patients’ antemortem microbiomes. (Fig. 8b). The proportion of newly acquired resistances in the rectal swab microbiomes was significantly lower, at about half the rate, 22.85%.

The potential association between antibiotic use, newly emerged resistances, and the corresponding healthcare-associated infection-causing species was also investigated (Fig. 8c and e). In oropharyngeal swab samples, *Pseudomonas aeruginosa* and *Enterococcus faecium* were identified as the primary species associated with the highest proportion of newly emerged resistances (81.26% and 16.7% of total), while *Escherichia coli* and *Stenotrophomonas maltophilia* also exhibited remarkable gain in the different NERs (Fig. 8c). For *P. aeruginosa*, the NERs were most frequently related to treatment with vancomycin in the form of combination therapy, and to a lesser extent, piperacillin-tazobactam. In instances of *E. faecium*, the development of resistances was predominantly observed against macrolides, fluoroquinolones, streptogramins, and glycopeptides, which are commonly linked with the use of vancomycin and meropenem treatments in combination therapy. In our patient cohort, *E. coli* was the most abundant species in both sample populations taken postadmission and antemortem (Fig. 8d and f). Its relative abundance showed a moderate decrease during the hospital stay (from OS PA rf: 0.33 to OS AM: 0.26, from RS PA: 0.40 to RS AM: 0.27) (Fig. 8d and f). Among the NERs, in oropharyngeal swab samples, macrolide was the most abundant (13.8%), followed by tetracycline (9.5%) and phenicol (8.3%) (Fig. 8c). Across our entire patient cohort, tetracycline was by far the most abundant drug class (19.01%) and showed no change between the antemortem samples and the postadmission samples (Fig. 8d). In rectal swabs, among the NERs, cephalosporin was by far the most abundant resistance (85.83%) with the largest portion of these resistances associated with *Escherichia coli*, followed by *Klebsiella pneumoniae*, and *Pseudomonas aeruginosa*, while others (glycopeptide: 4.03%, penam: 3.4%) were negligible in comparison (Fig. 8e). Tetracycline resistance was highest in rectal swabs similar to the oropharyngeal swab samples, with a moderate increase in its relative abundance observed in the antemortem samples (from PA rf: 0.22 to AM: 0.25) (Fig. 8f).

In RS *A. baumannii* has developed 15 new resistances to various antibiotics against cephalosporin, penam, phenicol, carbapenem, monobactam, aminoglycoside, penem, fluoroquinolone, diaminopyrimidine, lincosamide, rifampicin, glycylcycline, phosphonic acid, tetracycline, and macrolide. These resistances were mainly induced by medication with vancomycin, amikacin, and piperacillin-tazobactam (Fig. 8e).

## Discussion

Antimicrobial resistance represents a critical global health and economic threat, directly causing 1.27 million deaths in 2019 and contributing to 4.95 million fatalities worldwide^16^. In the European Union, AMR accounts for approximately 33,000 deaths annually, with over 670,000 AMR-related infections imposing an estimated €1.1 billion in additional healthcare costs^16,42^. Similarly, the United States reports around 35,000 AMR-associated deaths each year^42^. The escalating burden of AMR underscores the urgent need for intervention, particularly in healthcare settings where multidrug-resistant pathogens pose a substantial risk^39^.

The primary aim of this study was to investigate the interdependence of resistance patterns across anatomical sites in patients, healthcare staff, and various high-touch hospital environments to deepen our understanding of the transmission and accumulation of AMRs, particularly those associated with multidrug-resistant bacteria.

Identifying early microbial biomarkers could significantly improve prognostic assessment in critically ill patients, where rapid deterioration is common. To this end, a key objective of our study was to determine whether the microbial composition of admission swabs could serve as early indicators of severe dysbiosis and progression to sepsis. By comparing oropharyngeal and rectal samples collected at admission, we aimed to evaluate their prognostic potential and identify the sampling site that offers more reliable insights, ultimately informing future screening strategies and clinical monitoring protocols.

Finally, we also investigated the emergence of novel resistance mechanisms induced by antibiotic therapies, providing insights into how treatment protocols shape the evolution of resistant pathogens within ICU settings.

This study builds upon a previous investigation - a six-month longitudinal study conducted at Markusovszky University Teaching Hospital, Szombathely, Hungary, focusing on antimicrobial resistance dynamics in a clinical setting^35^. In contrast to the earlier study, which involved 12 patients, the current work expands the cohort to 20 by including eight additional ICU patients admitted during a five-month follow-up period in 2023, who later succumbed in the ICU. Importantly, this study also introduces new sample sources and analytical perspectives: in addition to the 65 postadmission and antemortem oropharyngeal and rectal swabs collected every three days (including on the day of death or 1–2 days prior), samples were also obtained from healthcare personnel (*n* = 8) involved in patient care and sample handling, and ICU environmental key surfaces (*n* = 23) including bedrails, sinks, taps, CRRT equipment, nurse stations, keyboards, phones, door handles, and patient rooms. Unlike our prior study, which focused broadly on microbial dynamics, this analysis centers specifically on antimicrobial resistance by investigating AMR transmission, persistence, prognostic biomarkers, and the role of the ICU environment and staff in transmission dynamics.

Due to the extensive use of systemic antibiotics, especially in ICUs, AMR and multidrug-resistant organisms can spread easily via healthcare personnel and the hospital environment^40^. Our findings revealed distinct clustering patterns in AMR genes and MDROs across ICU environments, patients, and staff, with the ICU environment showing the highest AMR read counts, reinforcing its role as a major resistance reservoir. These observations align with previous research demonstrating that high-touch hospital surfaces can act as intermediate ARO reservoirs, with transmission occurring via patients, medical staff, and visitors^43–45^. The oropharynx, as a highly exposed site and a known reservoir for pathogens, may play a pivotal role in environmental AMR acquisition^46^. Its microbiota is particularly vulnerable in ICU settings due to interventions such as mechanical ventilation, critical illness, and antibiotic use, all of which can disrupt oral microbial communities and favor MDRO colonization^46,47^. We observed a strong overlap in AMR patterns between environmental samples and oropharyngeal swabs from patients, which may reflect frequent microbial and AMR exchange via contact-based transmission routes. This overlap could suggest that the oropharynx may serve as a key interface for environmental AMR acquisition, likely influenced by the high-touch nature of facial areas and commonly shared surfaces, underscoring the ICU environment as a persistent reservoir of antimicrobial resistance.

The staff microbiota displayed significantly lower AMR gene abundance and diversity, yet shared key MDROs with patient oropharyngeal samples. The lack of unique antibiotic-resistant organisms in staff samples further suggests that while healthcare workers may facilitate the movement of pathogens, they are less likely to be the primary source of novel resistance. This aligns with findings that hospital surfaces are frequently contaminated with resistant pathogens, which can persist for long periods and are often transmitted via healthcare workers’ hands following contact with contaminated surfaces^44,45,48^. From a biomarker perspective, our analysis underscores the oropharynx as a crucial site for early detection of resistant pathogens, given its high overlap with environmental MDROs. Our findings also highlighted the oropharyngeal microbiota as a potential superior target for prognostic screening.

In contrast, rectal swabs showed weaker connectivity to environmental sources, suggesting that while MDROs may reach the gut, their colonization might be hindered by stronger microbial competition compared to the oropharynx. This reduced overlap could also imply that rectal AMR profiles may be shaped more by internal microbial or reservoir-specific dynamics than by direct environmental acquisition.

The exceptionally high prevalence of MDROs in the ICU environment, compared to patients (lower but still substantial) and staff (significantly lower), underscores the critical role of these habitats in resistance transmission. It highlights the need for a much stronger focus on their significance and the risks they pose, as well as the implementation of more effective environmental sanitation protocols.

Multidrug-resistant pathogens, particularly the ESKAPE group -*Enterococcus faecium*, *Staphylococcus aureus*, *Klebsiella pneumoniae*, *Acinetobacter baumannii*,* Pseudomonas aeruginosa*, and *Enterobacter spp.* - are major drivers of hospital-acquired infections^49^. The oral cavity has been recognized as a significant reservoir of resistance genes, and studies have shown that oral colonization by resistant bacteria can precede systemic infections and may facilitate their spread to other body sites or the environment^46,50^. Furthermore, saliva serves as an excellent culture medium, and oral biofilms can harbor pathogens for extended periods, further supporting the role of the oral microbiome in hospital-associated infections^46,50^. Notably, in our study, the distribution of HAI-associated AMR in environmental samples exhibited higher AMR frequencies compared to healthcare staff rectal swabs. Oropharyngeal swabs from patients harbored the highest AMR burden, further reinforcing their role as major reservoirs. Strikingly, the microbiomes of healthcare staff more closely resembled environmental samples than those of patients, suggesting potential transmission routes and distinct reservoirs within the ICU. This may be due to several factors: medical staff often use strict contact precautions when interacting with patients but may engage with environmental surfaces, like keyboards, door handles, and equipment, with less consistent protection^51,52^. Repetitive contact with shared surfaces across rooms, combined with limited exposure to individual patient microbiota, could explain the stronger alignment between staff and environmental microbiomes.

Quantitative analyses identified *Bacteroides fragilis* as the predominant AMR-associated species, followed by *Escherichia coli* and *Streptococcus pneumoniae*. Furthermore, the substantial AMR burden in *Pseudomonas aeruginosa* and *Staphylococcus aureus* underscores their roles as persistent nosocomial pathogens. Notably, *Streptococcus pneumoniae* was more prevalent in oropharyngeal swabs from healthcare staff, a trend that could likely be explained by its natural presence in the oral cavity^53^. In contrast, patient samples were dominated by *Escherichia coli* and *Pseudomonas aeruginosa*, organisms more frequently associated with healthcare-associated infections^49,54^. These findings might also suggest notable differences in microbial colonization and AMR transmission patterns between patient and staff microbiomes.

Several studies emphasize the importance of environmental reservoirs in infection prevention by assessing antibiotic-resistant organisms in ICUs^43,55^. Although ICU environments can vary significantly, our data consistently identified *Pseudomonas aeruginosa* as the most frequently isolated organism from ICU surfaces, underscoring its ability to persist in hospital environments, particularly on high-touch surfaces and equipment, indicating its relatively high resilience and adaptability^56^.

Identification and comprehensive analysis of common antibiotic resistances in ICU-associated microbiota were conducted, focusing on resistances with above-average prevalence in our cohort. Environmental samples demonstrated the most distinct composition of CAMR classes. Moreover, the CAMR distribution patterns in oropharyngeal swabs from healthcare staff closely mirrored those observed in environmental samples, which similarity is likely attributable to the prolonged exposure of healthcare workers to the hospital environment, particularly in the ICU where continuous contact with high-touch surfaces and medical equipment is common^44,45,48^. Another notable observation was that antemortem samples from both oropharyngeal and rectal swabs showed the least similarity to environmental samples. This may be due to the severely dysbiotic microbiomes of critically ill patients in their final stages, which are marked by more dynamic microbial shifts^57–59^. However, in terms of differences in resistance class distributions across sample groups, statistically significant differences weren’t observed.

Regarding the eight CAMR classes exceeding 5% relative frequency, tetracycline resistance emerged as the most prevalent, with a relative frequency slightly above 20%, showing a similar prevalence across both patient and staff samples. Macrolide, fluoroquinolone, lincosamide, and cephamycin resistances were notably higher in staff samples, indicating a less diverse resistance profile among healthcare workers. Moreover, the differences found between the groups resistance class distribution were statistically significant. This is noteworthy because common first-line antibiotics, like carbapenems and tigecycline, can lead to resistance against fluoroquinolones, lincosamides, and cephamycins^36^. Since healthcare workers in ICU environments are exposed to a wider range of antibiotics, they have a higher risk of accumulating these resistances and transmitting them to patients^40,60^. Regular surveillance of healthcare staff for specific antibiotic resistances, particularly to fluoroquinolones, lincosamides, and cephamycins, would be a critical step in monitoring and controlling the spread of the common antimicrobial resistance. Interestingly, oropharyngeal and rectal samples from individual patients were more similar to each other than to those from healthcare staff, suggesting coordinated microbial shifts within patients. This may reflect the influence of systemic factors such as antibiotic exposure. Notably, studies have shown a connection between the oral and gut microbiome, indicating that microbial communities at these distant sites can interact or respond similarly to host and environmental pressures^61,62^.

Our research also aims to provide insights into the relationship between antimicrobial resistance carriage and patient mortality by uniquely employing microbiome-based data from oropharyngeal and rectal swab samples. The Kaplan–Meier estimator was employed to clearly distinguish patient survival rates based on the duration of hospital stay, identifying a critical survival threshold at 10 days. This threshold stratified our patients into early-, and late-mortality groups. Based on these, through analyzing AMR read counts from two anatomically distinct sites -the oropharynx and rectum- our findings revealed an intriguing trend where total AMR counts were notably higher in rectal samples from patients exhibiting prolonged survival. This observation suggests a potential influence of microbial load on survival dynamics, consistent with previous studies that have linked microbiome alterations to mortality risk during critical illness^58,59^, although statistical significance was not reached. Furthermore, our research uniquely highlights microbial translocation dynamics between these distant anatomical locations, showcasing a higher prevalence of core AMR-carrying species in patients with longer survival, suggesting increased microbial exchange or translocation among patients associated with slower disease progression.

Our study highlights distinct antimicrobial resistance patterns and microbial shifts between early mortality and late mortality ICU patients. Specifically, EM patients exhibited a notable presence of species unique to OS samples, while LM patients demonstrated greater diversity in unique species within RS samples. The oropharyngeal microbiome in these patients showed a unique composition with a notable presence of AMR-carrying species, suggesting that the upper respiratory tract plays a pivotal role in early infections and systemic deterioration^50,63^. A stronger correlation between OS and RS microbiomes in EM patients further suggests a potential interaction between the gut and respiratory microbiota, possibly facilitating pathogen translocation and rapid clinical decline. In contrast, LM patients exhibited a greater diversity of unique AMR species in rectal swabs, indicating that the gut becomes a more prominent reservoir for resistant pathogens over time also suggesting that prolonged ICU stays create an environment conducive to AMR accumulation in the gut, increasing the risk of microbiome-associated complications like bloodstream infections or sepsis.

However, these findings should be interpreted in light of certain limitations. The smaller sample size, resulted by the division into survival-based subgroups, could reduce statistical power and may limit generalizability. Additionally, the clinical heterogeneity of the patients, including variability in comorbidities, pre-existing conditions, and antibiotic treatment regimens, introduces confounding factors that could independently influence microbiome composition and resistance profiles. While our findings reveal potentially important associations between AMR patterns and patient outcomes, larger, stratified cohorts are needed to validate these observations and disentangle the contributions of host factors, treatment regimens, and microbial dynamics in shaping AMR trajectories in critical care settings.

Growing evidence supports the potential of microbiota-modulating strategies - such as probiotics, prebiotics, or fecal microbiota transplantation (FMT) - in restoring microbiome balance and mitigating adverse outcomes, particularly in patients at risk of microbiome-associated sepsis^64^.

By identifying microbial taxa that significantly differs between EM and LM patients, we uncovered early prognostic biomarkers indicative of severe dysbiosis and rapid clinical deterioration, detectable from microbial swab samples collected shortly after ICU admission.

Our findings might also provide a valuable foundation for microbiota-modulating strategies, such as probiotics, prebiotics, or fecal microbiota transplantation (FMT), to mitigate dysbiosis and improve infection control, particularly in ICU patients prone to microbiome-associated sepsis. By reducing microbiome instability, these interventions could help prevent complications arising from microbial imbalances, ultimately enhancing patient outcomes^65,66^.

Through microbiome-based biomarker profiling, we aimed to establish an early prognostic stratification system that highlights distinct dysbiotic states, offering actionable therapeutic targets for immediate intervention. This approach advances precision medicine in critical care by enabling the early identification of high-risk patients who could benefit from intensified supportive care, personalized antimicrobial regimens, and tailored immunomodulatory therapies.

Our study also aims to provide insights into the potential role of multidrug-resistant organisms as early biomarkers predictive of sepsis development in ICU patients. Unlike previous research^67–70^, which has primarily focused on established bloodstream infections or culture-based pathogen identification, our study employs high-resolution metagenomic analyses to detect microbial shifts preceding sepsis onset. We identified four key MDRO species (*Listeria monocytogenes*, *Mycobacterium tuberculosis*, *Staphylococcus haemolyticus*, and *Streptococcus agalactiae*) significantly enriched in postadmission samples from patients who later developed sepsis, highlighting a potential window for early microbial-based risk assessment.

Among the identified MDRO species, *M. tuberculosis* exhibited the highest relative abundance. While *M. tuberculosis* is not typically associated with acute sepsis, its presence in ICU patients at risk for sepsis warrants further investigation, as it may indicate underlying immune dysregulation or serve as a sentinel marker for systemic inflammatory shifts.

Our study also uniquely characterizes the antimicrobial resistance profiles of sepsis-associated MDROs at a level of detail not commonly reported in ICU-based studies. While *M. tuberculosis* exhibited resistance determinants limited to macrolides and penams, *L. monocytogenes* showed a strong phenicol-dominated resistance profile, and *S. haemolyticus* and *S. agalactiae* displayed broader resistance mechanisms, including resistance to lincosamides and streptogramins.

Nosocomial infections represents a major threat to patients’ safety, ranking as the fifth leading cause of death among hospitalized individuals^41^. ICU patients face a significantly higher risk, up to tenfold, compared to general ward patients^71,72^. Although prophylactic antibiotics are vital in preventing severe infections, their widespread use raises concerns over accelerating antimicrobial resistance^73,74^. Selective pressure exerted by antibiotics promotes the expansion of gut bacteria carrying resistance genes, positioning the gastrointestinal tract as a key reservoir for AMR dissemination via horizontal gene transfer^75,76^. While the gut’s role is well-studied, the oropharyngeal microbiome remains underexplored, despite its proximity to infection sites and potential importance in resistance acquisition and transmission.

Notably, only a small fraction of rectal swabs exhibited newly emerged resistance compared to oropharyngeal swabs. In our study cohort, vancomycin and levofloxacin, a broad-spectrum fluoroquinolone, were the most frequently administered antibiotics, often in combination. The smaller fraction of newly emerged resistance in rectal swabs compared to oropharyngeal swabs could be explained by the applied regimes. Vancomycin and levofloxacin, both broad-spectrum antibiotics, are particularly effective against respiratory pathogens, exerting greater selective pressure in the oropharyngeal region^77,78^. Moreover, both vancomycin and levofloxacin are administered intravenously or orally, thus they may not reach the gastrointestinal tract in significant concentrations. As a result, their impact on the gut may be more limited compared to the oral microbiome^78,79^. This selective pressure likely contributed to the emergence of resistances in this area, as evidenced by the relatively high rates of MRSA observed in both patient oropharyngeal swabs and environmental samples.

While our study provides important insights into the dynamics of antimicrobial resistance in the ICU, there are some limitations to consider. The sample size, particularly for staff samples, was relatively small, which may limit the generalizability of some of our findings. Additionally, dividing the patient cohort into subgroups based on early and late mortality resulted in smaller sample sizes, which could influence the statistical power of certain comparisons. However, despite these limitations, our study also has several strengths. We took a comprehensive approach by examining not only patient samples from both oropharyngeal and rectal sites, but also samples from healthcare staff who had daily contact with the patients, as well as key ICU environmental samples. This broader perspective helps provide a more holistic view of AMR transmission and persistence in the ICU environment. Our findings therefore lay a solid foundation for future studies with larger sample sizes, which will be essential for further validating and expanding upon the patterns observed in this study.

## Conclusion

This study offers a unique, high-resolution analysis of antimicrobial resistance dynamics in the ICU through a longitudinal, multi-source sampling approach at Markusovszky University Teaching Hospital, Hungary. By systematically tracking AMR reservoirs and transmission pathways from admission to death, it integrates critically ill patients, healthcare staff, and the ICU environment, providing unprecedented insights into microbial exchange, resistance dissemination, and targeted intervention strategies in critical care settings.

Our key findings are as follows:

- The ICU environment exhibited the highest AMR burden, surpassing both patient and staff microbiomes, reinforcing its potential role as a major AMR reservoir.
- Strong genetic similarities between environmental AMR genes and patient oropharyngeal swabs may reflect frequent microbial exchange via contact-based transmission in the ICU setting.
- The oropharyngeal microbiome showed overlap with environmental-, staff-, and patient-derived MDROs, suggesting it might be a valuable prognostic target.
- Rectal swabs showed weaker connectivity to environmental sources, which could be attributed to the more competitive gut microbiota, that may hinder successful colonization.
- Healthcare staff microbiomes HAI-associated AMR profiles showed resemblance to environmental samples, suggesting that external contamination sources may contribute.
- Patients with prolonged ICU stays exhibited increased AMR accumulation in the gut, which could potentially raise the risk of bloodstream infections and sepsis.
- A key future research direction is microbiome-based prognostic screening, which could enable early identification of high-risk patients and support personalized antimicrobial interventions.
- In line with this approach, we identified four key MDRO species (*Listeria monocytogenes*,* Mycobacterium tuberculosis*,* Staphylococcus haemolyticus*, and *Streptococcus agalactiae*) enriched in early postadmission samples of patients who later developed sepsis, suggesting potential early biomarkers.
- Oropharyngeal AMR burden might correlate with early mortality, whereas gut-associated AMR could be more prominent in patients with prolonged survival, suggesting different AMR-driven disease trajectories.
- Vancomycin and levofloxacin, the most frequently administered antibiotics in this cohort, were associated with elevated resistance levels in the oropharyngeal microbiome, possibly because of the limited impact on the gut microbiota due to their intravenous administration.
- *Pseudomonas aeruginosa* and *Staphylococcus aureus* were the most persistent ICU-associated pathogens, highlighting their resilience in hospital environments.

## Materials and methods

### **Study design**,** population**,** and sampling**

A total of 96 metagenomic samples were analyzed, derived from three primary sources. The largest subset comprised 65 microbiome samples from 20 deceased ICU patients, including 33 rectal swabs (15 postadmission, 18 antemortem) and 32 oropharyngeal swabs (15 postadmission, 17 antemortem). Additionally, 8 samples were obtained from 4 healthcare workers (2 nurses, 2 doctors), consisting of 4 oropharyngeal and 4 rectal swabs. To further expand the scope, 23 environmental samples were collected from various ICU surfaces, such as bedrails, sinks, medical devices, and other high-touch areas.

This retrospective longitudinal cohort study was conducted in the Central Anesthesia and Intensive Care Unit of Markusovszky University Teaching Hospital, Szombathely, Hungary, between February 15 and June 22, 2023. It builds upon a previously published investigation^35^, expanding the patient cohort and incorporating environmental and healthcare worker sampling. While some patients overlapped with the prior study, this research introduces additional subjects and microbiome samples, providing a broader analysis of antimicrobial resistance dynamics in the ICU.

The patient cohort (*n* = 20, 13 males, 7 females, mean age 69.8 ± 9.9 years, median ICU stay 12.1 days, range 2–35) included 12 previously analyzed individuals and 8 new patients, enhancing statistical robustness. Inclusion required ICU admission for at least 48 h and in-hospital mortality. Sampling adhered to established protocols^35^, with rectal and oropharyngeal swabs collected at two critical time points: upon ICU admission (postadmission) and one to two days before death (antemortem). Patient samples were collected by the participating staff members, ensuring consistency in sampling techniques. Healthcare personnel were self-sampled by using the same procedure. Environmental sampling targeted high-contact surfaces such as handwashing facilities, hospital spouts, taps, medical equipment (Astrup devices, CRRT systems), nurse stations, telephones, ultrasound systems, rapid testing devices, storage rooms, and laboratories (details provided in **Supplementary File 1**). Environmental sampling was conducted twice during the study period, with each surface being sampled multiple times. The collected subsamples were then pooled together, resulting in a total of 23 environmental samples.

Patient demographic, biochemical, and clinical variables of interest were collected as patient-associated metadata, such as age, sex, and sepsis status. Additional variables associated with the follow-up, such as antibiotic administration, and outcome variables (ICU stay [days], complications, discharge condition), were also recorded.

Sepsis and septic shock diagnoses followed the Third International Consensus Definitions for Sepsis and Septic Shock. In this expanded cohort, 11 patients (55%) were diagnosed with sepsis, and 10 progressed to septic shock. Patients or their relatives were personally informed of the study in the presence of a responsible clinician and sampling was carried out upon written consent by the patient or a legal representative. The study was approved by the institutional review board of Markusovszky University Teaching Hospital Regional Scientific and Research Ethics Committee (Ethical permission number: 4/2023). All methods were performed in accordance with the Declaration of Helsinki. Written informed consent was obtained from all participants for the collection of medical data and the use of samples for research purposes.

### Nucleic acid extraction

DNA extraction from swab samples was performed using the DNeasy^®^ PowerSoil^®^ Pro Kit (Qiagen, Germany, Cat. 47016) following the manufacturer’s instructions. Minor modifications were made to optimize the DNA extraction. In brief, 300 µl of supernatant was transferred into PowerBead Pro Tubes and incubated at 65 °C for 10 min. With the use of a MagNA Lyser Instrument (Roche Applied Sciences, Germany), samples were lysed two times at 3,000 × g for 30 s. Finally, 70 µl of Solution C6 was added and incubated at room temperature for 5 min before centrifugation. DNA concentrations were determined fluorometrically using a Qubit^®^ Fluorometric Quantitation HS dsDNA Assay (Invitrogen by Thermo Fisher Scientific, Cat. 2600187) kit on a Qubit^®^ 4.0 Fluorometer (Thermo Fisher Scientific, USA).

### **Library preparation**,** sequencing**,** and metagenomic data processing**

The genomic DNA was randomly sheared into short fragments at Novogene Bioinformatics Technology (Beijing, China). The obtained fragments were end-repaired, A-tailed, and further ligated with Illumina adapters. The fragments with adapters were size-selected, PCR amplified, and purified. The library was checked with Qubit and real-time PCR for quantification and bioanalyzer for size distribution detection. Shotgun sequencing was conducted on an Illumina NovaSeq 6000 instrument (Illumina, USA) with a 150-bp paired-end sequencing run at Novogene Bioinformatics Technology (Beijing, China). The sequencing yielded a minimum of 20 million reads per sample. To ensure the availability of 20 million reads per sample, each sample was re-extracted and purified as needed until the required purity (OD260/280 = 1.8–2.0) and concentration (≥ 10 ng/µL) were achieved for each metagenomic isolate. Prior analysis samples quality were checked using FASTQC^80^. Microbial analysis was completed using the SqueezeMeta pipeline (v1.6.3) utilizing the co-assembly option with no binning^81^. Briefly, paired-end reads were assembled using Megahit before taxonomic and functional annotation using the DIAMOND v.2.19 sequencing aligner to the GenBank^82,83^. Contigs were evaluated by mapping reads back to the co-assembled sequences to assess coverage and support downstream analyses. The KIFÜ Hungarian High-Performance Computing Competence Center (HPC CC) Komondor HPC was used with 48 CPU cores and 90 GB RAM per sample. For antibiotic resistance bioinformatics analysis KneadData software was performed to quality control on sequencing data, using Trimmomatic and Bowtie2^84–86^. To predict antibiotic resistome RGI software was used with CARD database^87,88^.

### Description of shotgun sequencing results

Shotgun metagenomic sequencing was carried out on Illumina NovaSeq platform (for reads for each sample, see **Supplementary File 4**). After quality filtering with the Trimmomatic software, 39,289,881 ± 11,782,090 average reads per sample were obtained. Read counts differed significantly across sample sources. Patient samples had the highest average read counts, which were significantly greater than those from the environment (Wilcoxon rank-sum test; Patient mean: 55,558,321 vs. Environment mean: 31,271,285; *p* < 0.0001) and healthcare personnel (Wilcoxon rank-sum test; Patient mean: 55,558,321 vs. Staff mean: 47,005,143; *p* = 0.025). While environmental samples had lower average read counts than staff samples, this difference was not statistically significant (Wilcoxon rank-sum test; Environment mean: 31,271,285 vs. Staff mean: 47,005,143; *p* = 0.1).

### Statistical analysis and data visualization

Continuous variables were expressed as mean ± standard deviation. The Wilcoxon rank-sum test was used to compare continuous variables. All statistical tests were two-tailed, and a *P* < 0.05 value was considered statistically significant. The graphs were made with the R package ‘ggplot2’ (version 3.5.0)^89^. Heatmaps were constructed with pheatmap R package (version 1.0.12)^90^. Venn diagrams were performed with limma R package (version 3.60.3)^91^. To examine the differences in MDRO structures between groups, PCoA was conducted using the vegan v.2.6-4 package in R^92^. To assess the survival probability of the patients Kaplan Meier plot was made by using the survival and survminer R packages (version 3.8-3 and version 0.5.0)^93,94^. Roc curves were used to define the Area under the curve (AUC) with the pROC R package (version 1.18.5)^95^. Network analysis of different antibiotic combinations throughout the study was made using igraph v.1.5.0.1 R package^96^. To identify the distribution pattern of newly acquired resistance classes and their association with healthcare-associated infection (HAI)-causing species, a Sankey diagram was generated using the R package NetworkD3 v.0.4^97^. MDS plots with linear regression lines were performed by using vegan v.2.6-4 package in R^92^. LefSe score was calculated using microbiomeMarker R package^98,99^.

Patients were stratified based on specific clinical criteria relevant to each analysis. All patients were treated as a single cohort to capture overarching trends for general assessments of AMR distribution across sample types and sources (e.g., oropharyngeal vs. rectal swabs, environment, staff). In analyses focused on survival, patients were categorized into early mortality (EM) and late mortality (LM) groups based on a survival threshold of 10 days, as determined by Kaplan–Meier analysis. For investigations specifically examining the impact of sepsis, comparisons were made between patients who developed septic shock and those who did not, independent of survival duration. This targeted stratification approach was chosen to address different research questions without introducing unnecessary model complexity. However, we acknowledge that not all clinical variables were adjusted for simultaneously in each analysis, and interpretations were made within the context of the specific stratification applied.

## Electronic supplementary material

Below is the link to the electronic supplementary material.

Supplementary Material 1

Supplementary Material 2

Supplementary Material 3

Supplementary Material 4

## Data Availability

All sequence data used in the analyses were deposited in the Sequence Read Archive (SRA) (http://www.ncbi.nlm.nih.gov/sra) repository, under PRJNA1100796.
