# Supplementary material for: Analysis of ICU resistome dynamics in patients, staff and environment for the identification of predictive biomarkers of sepsis and early mortality
Source: Sci Rep. 2025 Jul 11;15:25080. doi: 10.1038/s41598-025-10848-8 (PMC12254246; doi:10.1038/s41598-025-10848-8)
Supplement: Supplementary file 2 — Supplementary Material 2 [file 41598_2025_10848_MOESM2_ESM.docx]

The mean relative frequency of the antimicrobial resistances (AMRs) was calculated, which was 0.00046. Then in each group common antimicrobial resistances (CAMRs) displaying an above-average relative occurrence were identified (**Supplementary Figure 1**).


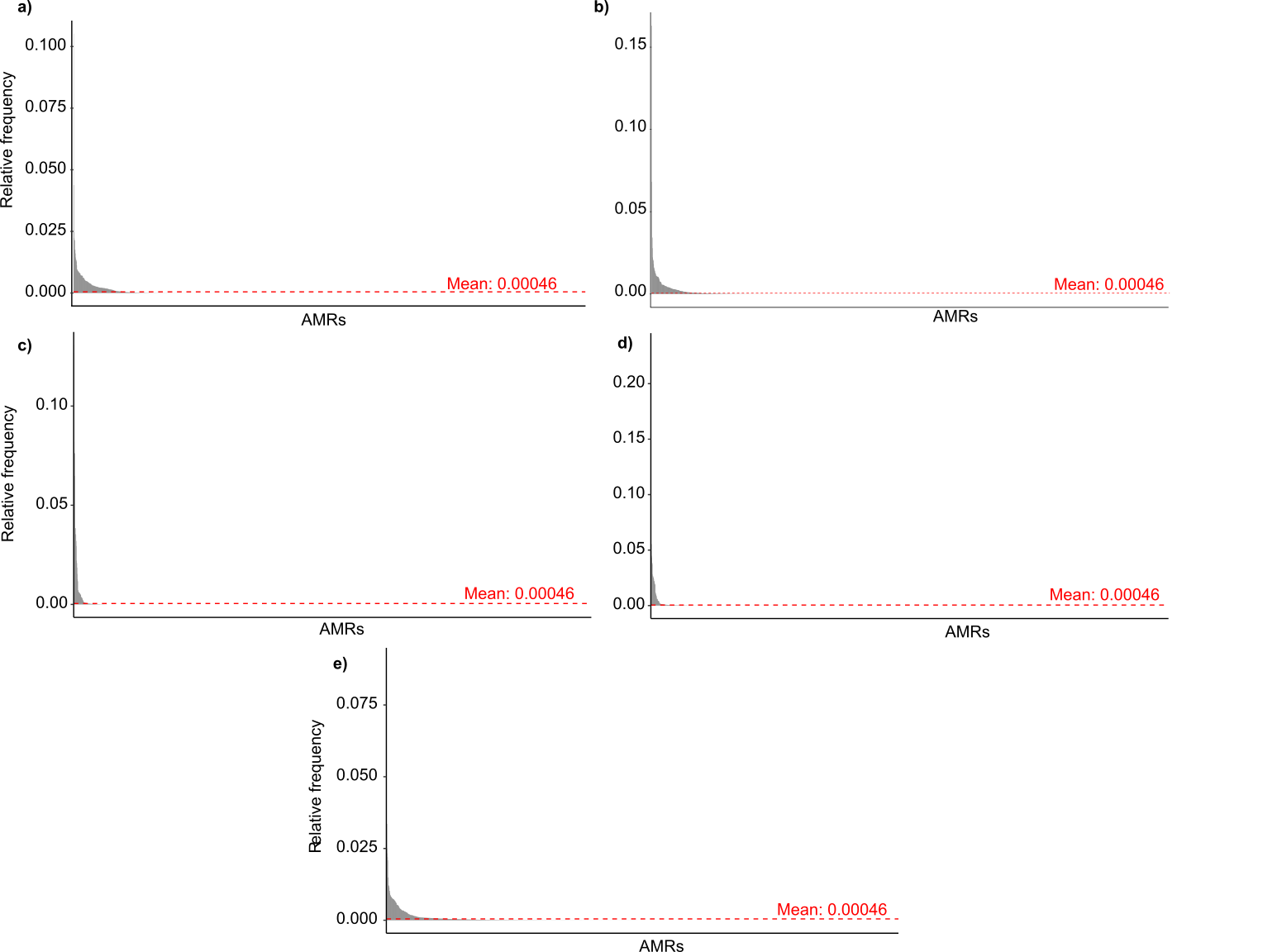


**Supplementary Figure 1.** **The common antimicrobial resistances (CAMRs) in our study groups.** Barplots show the CAMRs in the patients' a) oropharyngeal and b) rectal swab samples, in the staffs’ c) oropharyngeal and d) rectal swab samples, and e) in the environment. Red dashed line indicates the mean relative frequency of the AMRs.
